# Sensory Afferent Neural Circuits Mediate Electroacupuncture to Improve Swallowing Function in a Post‐Stroke Dysphagia Mouse Model

**DOI:** 10.1111/cns.70514

**Published:** 2025-07-24

**Authors:** Yong Dai, Jiahui Hu, Qianqian Wang, Jia Qiao, Yueqin Tian, Chao Li, Jiemei Chen, Fei Zhao, Xinya Li, Chunyan Liu, Ruihuan Pan, Haining Ou, Nenggui Xu, Hongmei Wen, Zulin Dou, Qiuping Ye

**Affiliations:** ^1^ Department of Rehabilitation Medicine Third Affiliated Hospital of sun Yat‐Sen University Guangzhou China; ^2^ Department of Rehabilitation Medicine Guangdong Provincial Hospital of Chinese Medicine Guangzhou China; ^3^ Clinical Medical College of Acupuncture‐Moxibustion and Rehabilitation Guangzhou University of Chinese Medicine Guangzhou China; ^4^ School of Public Health and Management Guangzhou University of Chinese Medicine Guangzhou China; ^5^ The Second Clinical Medical School of Guangzhou University of Chinese Medicine Guangzhou China; ^6^ Acupuncture and Moxibustion Deparment Guangdong Provincial Hospital of Chinese Medicine Guangzhou China

**Keywords:** electroacupuncture, Lianquan acupoint (CV23), neural circuit, post‐stroke dysphagia, sensory afferent

## Abstract

**Background:**

Electroacupuncture (EA) has been reported to improve post‐stroke dysphagia (PSD) effectively. However, the underlying afferent neural circuit and neurological mechanism involved in improving PSD remain poorly understood.

**Methods:**

A PSD mouse model was established via the photochemical embolization method. Laser scatter contrast imaging was used to evaluate blood perfusion. Videofluoroscopic swallowing study, flexible endoscopic evaluation swallowing, and electromyography were used to assess the swallowing function. Neuronal activities and neuron types were detected by immunofluorescence. Synaptic connections between the nucleus tractus solitarii (NTS), the ventral posteromedial thalamic nucleus (VPM), and the primary sensory cortex (S1) were verified by neural tracing. Finally, photogenetic, chemogenetic, and in vivo electromyography or electrophysiological records were used to explore the possible afferent neural circuits of EA therapy for PSD.

**Results:**

EA treatment potentiated the blood perfusion of CV23 and S1, improved the area under the curve, pharyngeal transit time, and vocal fold mobility in PSD model mice. EA also activated neuronal activities in VPM, while chemical genetic inhibition of VPM attenuated the swallowing function of EA enhanced in PSD mice. Neural tracing revealed the presence of direct synaptic connections in the neural circuit of NTS‐VPM‐S1, and excitatory neurons were the predominant type of synaptic projection. Activation of this circuit improved the swallowing function in PSD mice, whereas its inhibition impaired the swallowing function; this effect was reversible by EA‐CV23.

**Conclusion:**

Our findings uncover the importance of sensory afferent neural circuits NTS‐VPM‐S1 in driving the protective effect of EA‐CV23 against dysphagia and thus reveal a potential strategy for PSD intervention.

## Graphical abstract

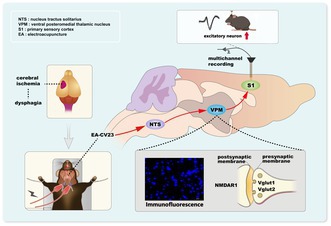

**Abbreviations**
AUC: area under curve
CNO: clozapine n‐oxide
CV23: lianquan acupoint
EA: electroacupuncture
ETT: esophageal transit time
FEES: flexible endoscopic evaluation of swallowing
HSV: herpes simplex virus
ISI: inter swallow interval
LSCI: laser speckle contrast imaging
M1: primary motor cortex
MMAR: mean motion angle ratio
MMRR: mean motion range ratio
NMDAR1: N‐methyl‐D‐aspartic acid receptor
OTT: oral transit time
PSD: post stroke dysphagia
PTT: pharyngeal transit time
RV: rabierabies virus
S1: primary sensory cortex
VFSS: video fluoroscopic swallowing study
Vglut1: vesicular Glutamate Transporter 1
Vglut2: vesicular Glutamate Transporter 2
VPM: ventral posteromedial thalamic nucleus

## 1 Introduction

Stroke is the second most common cause of death worldwide and is the most common cause of post‐stroke dysphagia (PSD). In terms of the type, severity, and assessment of dysphagia, the reported incidence of PSD is as high as 80% [1, 2]. Persistent dysphagia independently predicts hospitalization and other adverse outcomes, such as physical pain, malnutrition, weight loss, aspiration, and even death [3, 4]. Furthermore, PSD may also cause a decline in the social ability of patients [5]; those complications make the treatment of dysphagia a focus of rehabilitation.

The current therapeutic methods for PSD mainly consist of enhancing the swallowing motor output or sensory afferents, of which the treatments to increase sensory afferents include pharyngeal electrical stimulation (PES), neuromuscular electrical stimulation (NMES) [6], oral sensory stimulation, and electroacupuncture (EA), etc. Among them, PES and EA are commonly used to treat dysphagia. PES, as a novel rehabilitation treatment for neurogenic dysphagia, acts by placing specific catheter electrodes in the posterior pharyngeal wall, which might be uncomfortable for some patients. The act mechanism of PES may be associated with the activation of sensation within the mucosa and the transmission of sensory information to the NTS as well as higher swallowing centers [7]. Compared to PES stimulation, EA only needs to target the sensory receptors in the submandibular region in the front of the neck through a needle and transmits the sensation to the center through the vagus nerve, which has the advantages of simple operation, high safety, affordable price, and accurate efficacy. EA was widely used to promote the recovery of both sensory and motor functions, while PES may help facilitate extubation and improve dysphagia in patients undergoing tracheotomy [8]. As a method of peripheral stimulation, EA has been applied in several fields including PSD and has been proven to be effective and safe, with Lianquan acupoint (CV23) being the most frequently used [9]. Our previous studies reported that EA‐CV23 can improve swallowing function in PSD model mice through the activation of primary motor cortex (M1) inputs to the nucleus tractus solitarii (NTS) through the parabrachial nucleus (PBN), and the M1‐PBN‐NTS neural circuit was the major motor efferent pathway mediating the ability of EA‐CV23 to improve swallowing in PSD mice [10]. However, the previous study aimed to observe the effects of EA on swallowing movements in terms of motor cortical output, but the sensory afferent input mechanism of EA‐CV23 was not fully understood.

Swallowing involves a complex network of cortical and subcortical centers, among which the swallowing central pattern generator (sCPG) plays an important role in regulating swallowing actions and sensations. The sCPG has been recognized as the first relay station for gustatory and visceral afferent information [11, 12, 13, 14], among which NTS is a key element that receives informational afferents and uploads them to higher sensory cortices, including gustatory, tactile, and other deep sensory inputs [15]. The primary sensory cortex (S1) is considered the highest integration center of somatosensory, receiving projections from the thalamus and integrating sensory information from different parts of the body [16]. Magnetic resonance imaging (MRI) revealed that S1 was significantly activated during swallowing [17, 18], and S1 activation was significantly reduced in patients with functional dysphagia [19]. The ventral posteromedial thalamic nucleus (VPM), located in the ventral part of the thalamus, receives neuronal inputs from the medial thalamic system and the thalamic trigeminal tract, and then projects directly to S1 [20]. Many reports have shown that the VPM is responsible for receiving sensory information from certain areas of the face and regulates taste, whose neuronal signals from the gustatory system may act as relay nuclei for peripheral sensory afferents to higher center [21]. These findings collectively suggest that the NTS, VPM, and S1 may interact to regulate swallowing function. However, there is no direct evidence illustrating how these brain regions jointly participate in the regulation of swallowing function.

In this study, PSD model mice were subjected to EA‐CV23 intervention 24 h after modeling, and their swallowing function was assessed in combination with videofluoroscopic swallowing study (VFSS), flexible endoscopic evaluation swallowing (FEES), and electromyography (EMG). On the basis of neuromodulatory strategies such as chemical genetics and optogenetics, we propose the hypothesis that the NTS‐VPM‐S1 neural circuit mediates the treatment of PSD by EA‐CV23, exploring the sensory afferent mechanisms of EA‐CV23 and the role of VPM in swallowing, which will provide further mechanistic evidence for EA‐CV23.

## 2 Materials and Methods

### 2.1 Ethics Statement

All experimental procedures were performed following the National Institutes of Health Guide for the Care and Use of Laboratory Animals and were approved by the Committee for Care and Use of Research Animals of Guangzhou University of Chinese Medicine (No. 20221011004), which conforms to the guidelines for the care and use of animals established by that committee.

### 2.2 Animals

Male C57BL/6 mice (6–7 weeks, 20–25 g) were selected and purchased from Guangdong Medical Animal Center (SCXK (GD) 2022–0002). All the mice were housed in the SPF‐grade laboratory animal room of the South China Research Center of Acupuncture and Moxibustion (SYXK (GD) 2022–0179). The rearing environment included a temperature of 23°C–26°C, 50%–60% relative humidity, and a 12 h light/12 h dark cycle, and the animals had free access to food and water. The padding used for the animals was changed twice a week, and the animals were acclimatized for at least 1 week before the experiment. A total of 176 mice were included in the statistical analysis after excluding accidental deaths, and each mouse was randomly grouped by numerical table method.

### 2.3 PSD Modeling

After intraperitoneal injection with 0.2 mL of 1.5% Bengal rose solution for approximately 10 min, mice were placed in a mixed oxygen/air anesthesia induction chamber with 4% isoflurane until anesthesia was achieved. The mice were subsequently transferred to an anesthesia mask and maintained with 2% isoflurane supplied by an animal anesthesia machine (R500, RWD, China). A 532 nm laser beam with a diameter of 1 mm was used to illuminate the right M1 cortex (AP: −0.12 mm, ML: −1.03 mm) for 6 min. Detailed descriptions can be found in our earlier work [10].

### 2.4 Laser Speckle Contrast Imaging (LSCI)

The cerebral blood perfusion was examined by a Laser Speckle Blood Monitor (PSI‐ZR, Pericam PSI, Sweden) as previously described [10]. The mice were anesthetized with 2% isoflurane in an oxygen/air mixture and positioned on a stereotaxic apparatus prior to making a paramedian incision to access the skull bone to detect the blood perfusion in the S1 and CV23 (area: 4 mm^2^). During the procedure, laser speckle imaging was performed with a sampling frequency of 5 Hz, resolution of 1386 × 1034 pixels. The scanning lasted for 120 s. The variation of blood perfusion was analyzed with the PIMsoft, and the average value in blood perfusion units was calculated.

### 2.5 EA–CV23 Procedure

Mice were subjected to EA treatment 24 h after PSD modeling as previously described [10]. A stainless‐steel needle (Suzhou Medical Appliance Factory, China) was used at a depth of 0.5 cm into the CV23 acupoint by obliquely inserting it, which is located at the presumed mylohyoid. Electrical stimulation was performed via a HANS‐200A stimulator (HANS200/00BEA, HANS, Beijing, China) with a stimulation strength of 1 mA, a continuous wave, and a frequency of 2 Hz, and the treatment duration was 15 min each day over a period of 3 days. After finishing the treatment, the mice were placed on an electric blanket to be awakened. Anesthesia was induced with 2% isoflurane in all the PSD + EA group, the PSD group, and the normal group, but only the EA group received EA stimulation, and the remaining two groups did not undergo EA stimulation.

### 2.6 VFSS Procedure and Video Analysis

VFSS was conducted to assess the swallowing function. The VFSS was always conducted at least 6 h before laryngoscopy to avoid confounding effects of anesthesia (required for laryngoscopy) on VFSS outcomes.

In this study, a customized VFSS device (PUAI Medical, Zhuhai Pulide Medical Equipment Co. Ltd., Zhuhai, China) included an X‐ray device with a high sampling rate and produced a comfortable, low‐stress, self‐feeding examination environment to assess the swallowing function of the mice. Behavioral conditioning was performed 48 h prior to the VFSS test to ensure that the mice were familiar with and accepted the special food containing iodohydrin (1440, GE Pharmaceuticals (Shanghai) Co.) and the test environment. The special food was prepared by mixing 1 mL of water and 1 mL of iohexol with 10 g of maintenance food. Mice were restricted from fluid/food intake for 12 h prior to VFSS. During the test, mice were individually enclosed in a customized chamber. Each mouse was then exposed to a low dose of radiation for approximately 3 min to assess the phasing of swallowing and bolus area during free‐feeding. To minimize radiation exposure, the fluoroscope was activated only when the mice were eating. Fluoroscopic videos were captured at 30 fps during real‐time viewing via a computer monitor. The recorded data were imported into the analysis software (LGT‐5500 Swallowing Image Digital Acquisition and Analysis System). The swallowing process was subsequently analyzed frame by frame, and the analysis indices included the interswallow interval (ISI), oral transit time (OTT), pharyngeal transit time (PTT), esophageal transit time (ETT), and area of the bolus in one mouthful [22, 23].

Videos with AVI files were subsequently analyzed frame by frame by three independent reviewers who were unaware of the grouping (Jiahui Hu, Qiuping Ye, Yueqin Tian). The primary analyst viewed each video, identifying and analyzing three to five consecutive swallow cycles (approximately 10 s). The secondary analyst independently analyzed three to five measurements of each swallowing parameter for each mouse initially identified and analyzed by the primary analyst. The third analyst determined the consistency of each swallowing parameter. All differences were reanalyzed to achieve 100% agreement. The average of three to five concordant values for each swallowing parameter for each mouse was statistically analyzed. When fewer than three measurements were obtained for a single swallowing parameter for a given mouse, the missing values were entered into the statistical database for the corresponding swallowing parameter.

### 2.7 FEES Procedure and Video Analysis

To assess vocal folds (VF) mobility, FEES was performed according to an established protocol [24]. The mice were fasted for 4–6 h before the test to avoid food retention in the pharynx. An animal laryngoscope (XinSoft Information Technology Co. Ltd., Shanghai, China) and 1.25% tribromoethanol were prepared. When the mice were anesthetized, they were fixed in an ear bar in a supine position and spontaneously breathed. The mouse tongue was gently secured externally with a cotton swab, and then a micro‐operator‐controlled endoscope with a customized laryngoscope sheath was gently inserted into the oral cavity to observe the movement of the VF. Notably, the endoscope advanced slowly until the bilateral VFs filled the entire field of view, recording VF movement at 30 fps for approximately 3 min during spontaneous breathing. In mice, VF movement is spontaneous with respiration rather than induced. The assistant paid close attention to the state of the mouse, and when the mouse appeared to have shortness of breath and cyanosis of the tongue, the operator was reminded to promptly abort the laryngoscopic procedure [25].

**FIGURE 1:**
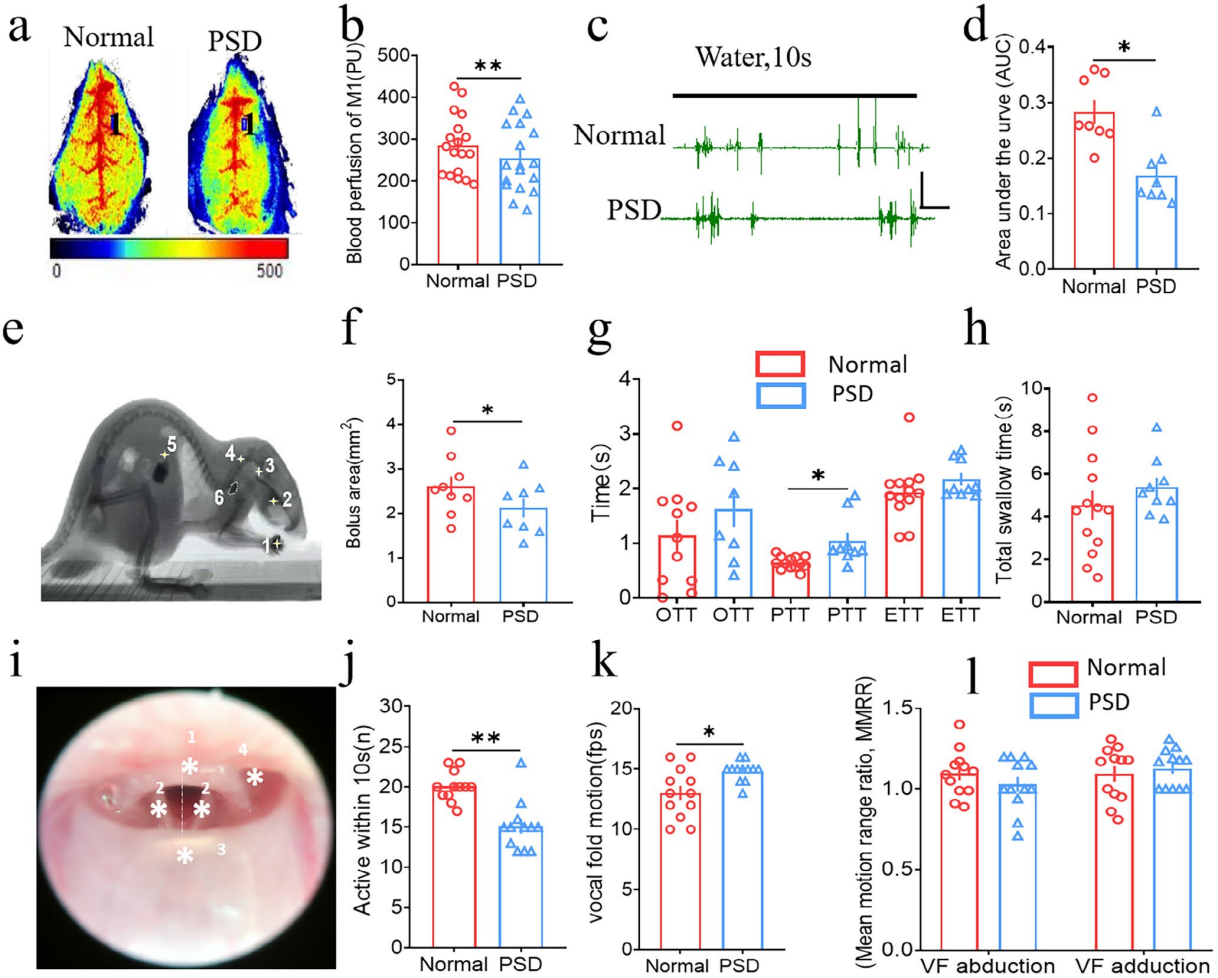
Swallowing function was impaired in a PSD mouse model induced by photothrombotic ischemia in M1. (a) Representative figure of the blood perfusion of M1 in the normal and PSD groups. Duration: 120 s, target area = 4 mm^2^. (b) Blood perfusion variation of M1 in the PSD group decreased compared with the normal group (Two‐tailed Student's unpaired *t* test, *t* = 3.094, *n* = 18, **p < 0.01, normal vs. PSD). (c) Representative example of the EMG in the normal and the PSD group. Black line: Water administration for 10 s with a velocity of 2 μL/s. Scale: 0.2 mV, 1 s. (d) EMG of mylohyoid decreased after PSD modeling in the PSD group compared with the normal group (Two‐tailed Student's unpaired *t* test, *t* = 5.287, *n* = 18; *p < 0.05, normal vs. PSD). (e) Representative example of VFSS. The meaning of plug signs and numbers: 1. bolus; 2. middle tongue; 3. pyriform crypt; 4. second cervical vertebra; 5. stomach; and 6. bolus area. (f) Bolus area was significantly smaller in the PSD group than in the normal group (Two‐tailed Student's unpaired *t* test, *t* = 3.837, normal *n* = 13, PSD *n* = 9, *p < 0.05, normal vs. PSD). (g) Results of OTT, PTT, and ETT between the two groups. PTT showed longer in the PSD group than in the normal group (Mann–Whitney U test, *Z* = −3.185, normal, *n* = 13, PSD, *n* = 9; *p < 0.05, normal vs. PSD). (h) No significant differences were seen in the comparison of total swallow time between the normal and the PSD groups (Two‐tailed Student's unpaired *t* test, *t* = −1.237, normal, *n* = 13, PSD, *n* = 9, *p* > 0.05). (i) Representative example of pharyngeal luminal structures of mice under FEES. The asterisk and numbers indicated: 1. epiglottic cartilage; 2. vocal folds; 3. root of the tongue; 4. pyriform crypt. The motion midline, left line (LL), and right line (RL) were tagged by white line. (j) Number of vocal fold activities within 10 s was significantly lower in the PSD group than in the normal group (Mann–Whitney U, Z = −3.833, *n* = 12; **p < 0.01, normal vs. PSD). (k) Time required for a single adduction and abduction in the PSD group was significantly longer than that in the normal group (Mann–Whitney U, Z = −1.436, *n* = 12, *p < 0.05, normal vs. PSD). (l) Ratio of the adduction angle to the abduction angle between the normal group and the PSD group was not significantly different. Data are shown as mean ± SD.

The videos of the MP4 files were viewed frame‐by‐frame by two independent reviewers (Jia Qiao and Qianqian Wang) via blinded video‐editing software (ImageJ software), and the heterogeneity was compared by a third reviewer (Jiemei Chen). If significant differences existed, they were discussed and reanalyzed with a meeting. ImageJ software was used to analyze VF motion dynamics. A 10 s clip was selected from each video recording on the basis of adequate visualization of the VFs and no aberrant camera movement. Then, three to five pictures of the maximum abduction and adduction of the VF in the video were intercepted, and a pair of points was manually placed on each VF (VF‐glottal region boundary) for tracking. Point selection was based on the anatomy of the VFs, which were selected on the upper (i.e., ventral) half of each VF to ensure greater sensitivity to small VF movements (Figure 1j). The mean motion range ratio (MMRR), number of vocal fold activities in 10 s, and duration of vocal fold activity per cycle (frames) were used to measure the differences in motion behavior between the left and right VFs to characterize the frequency and amplitude of VF motion [23].

### 2.8 In Vivo EMG Recording

As described in our previous publications [10], EMG recording was used to assess neuromuscular function by measuring the muscle response to nerve stimulation. To capture the EMG signal of the mylohyoid in awake mice, the mice were first anesthetized with isoflurane induction, removed from the anesthesia box, and quickly fixed in the supine position on a mouse adapter; the limbs were then secured using medical tape to prevent them from breaking free during the test. A pair of customized needle electrodes (approximately 15 mm in diameter) was then inserted unilaterally through the skin into the left hyoid bone for approximately 0.5 cm, and a PE tube (0.5 × 0.9 mm) for water delivery was placed on the hard palate of the mouse. Water (20 μL) was infused with a microinjection pump at a consistent rate of 2 μL/s for 10s, and the water was administered at intervals of at least 60 s to ensure that the mice had sufficient time to swallow the water. All EMG signals were acquired via Spike2 software (CED, UK). The data were digitized with a Power 1401 digitizer (CED, UK) at 5 kHz and bandpass filtered with a 1902 differential AC amplifier (CED, UK) at 0.1–1 kHz. Analysis was performed by calculating the area under the curve (AUC) within a 10‐s window after the onset of water delivery [26, 27].

### 2.9 Immunofluorescence (IF)

The mice were deeply anesthetized with 1.25% tribromoethanol and transcardially perfused with 25 mL of saline, after which the brain tissue was fixed with 25 mL of 4% paraformaldehyde (PFA). Whole brains were removed, placed in 4% PFA solution, and refrigerated at 4°C overnight. The samples were gradient dehydrated in 15% and 30% sucrose solutions, followed by optimal cutting temperature (OCT) compound embedding. Brain specimens were sliced with a cryotome to 40 μm. Then, the brain sections were rinsed three times with 0.01 M PBS for 5 min and blocked with a blocking mixture consisting of 1% BSA (Macklin, B885114, China) and 0.3% Triton X‐100 (Biosharp, BL935A, China) for 1.5 h at 37°C. The samples were then incubated for 2 days at 4°C. To detect the expression of c‐Fos, N‐methyl‐D‐aspartate‐receptor1(NMDAR1), Vesicular Glutamate Transporter 1(Vglut1) and Vesicular Glutamate Transporter 2(Vglut2), the primary antibodies used were as follows: anti‐NMDAR1: Abcam, EPR2481, 1:500; Vglut1: Affinity Biosciences, DF13657, 1:500; anti‐Vglut2: Abcam, ab79157, 1:500; rabbit anti‐c‐Fos: Synaptic Systems, 226,008, 1:500; and mouse anti‐c‐Fos: Abcam, EPR21930‐238, 1:500. The brain sections were incubated with secondary antibodies, namely, anti‐mouse‐conjugated Alexa Fluor 488 (Life, A21202, 1:500), anti‐rabbit‐conjugated Alexa Fluor 488 (Invitrogen, a11034, 1:500), and anti‐rabbit‐conjugated Alexa Fluor 594 (Life Technologies, a21207, 1:500), for 1 h at 37°C. The sections were subjected to nuclear staining with a DAPI solution at a concentration of 1 μg/5 mL for 5–10 min. A confocal microscope (Nikon, Japan) was used to visualize the samples at 20X or 40X magnification. The images were analyzed by an operator who was blinded to the process of the experiment. High‐resolution images of the S1 and VPM region were obtained at 60×, using a brain atlas (3rd ed., The mouse brian) for guidance. Area and positive cell counts were measured using ImageJ software (1.52a, National Institutes of Health, USA). The density of positive cells per section was calculated by dividing the total number of cells sampled by the total volume of the region at 60×. The expression of c‐Fos was manually quantified in different brain regions by an observer blinded to the experimental conditions [26].

### 2.10 Virus‐Mediated Anterograde Trans‐Synaptic Neural Tracking

The HSV‐EGFP virus was stereotactically injected into the NTS (AP: −6.48 mm, ML: −1.20 mm, DV: −4.43 mm), and the immunosuppressant bortezomib was injected intradermally with 0.2 mL immediately after injection. After 24, 48, and 72 h, the brain tissue was removed, followed by fixation of the brain tissues in 4% PFA overnight and 15% or 30% gradient dehydration. The brain tissues were sliced into 40‐μm slices via a cryosectioner (HM525, Thermo, USA) and imaged via confocal laser scanning microscopy (Nikon, Japan).

### 2.11 Recombinant Rabies Virus (RV)‐Based Retrograde Trans‐Synaptic Tracing

To explore the direct synaptic connections of the NTS‐VPM‐S1 neural circuit, S1(AP: +0.75, ML: −1.68, DL: 2.4) was transfected with retrograde tracer virus (rAAV‐CaMKIIa‐Cre‐WPRE‐hGH pA, retro), and the VPM was transfected with a mixed helper virus (rAAV2/9‐Ef1a‐Dio‐0RVG‐WPRE‐pA and rAAV2/9‐EF1G‐DIO‐H2B‐EGFP‐T2A‐TVA‐WPRE‐hGH‐pA to 1:1). After 14 days, RV‐EnvA‐ΔG‐dsRed was injected into the VPM (AP: −1.46, ML:−1.9, DL:3.75). The RV, whose glycoprotein (G) gene is deleted from the genome (RV‐ΔG), cannot spread across synapses, but G complementation enables the transsynaptic spread of RV‐ΔG to presynaptic neurons. The RV particles were packed with the fusion protein of the envelope protein (EnvA) of the recombinant avian sarcoma virus, which can specifically recognize the EnvA receptor TVA to infect cells. With the aid of helper viruses, RV‐EnvA‐ΔG can be transmitted retrogradely to upstream neurons. The mice were allowed to recover for 7 days and then were transcardially perfused with 0.9% saline and 4% PFA. The tissues were fixed with 4% PFA overnight and dehydrated with 30% sucrose solution for 3 days. The brain tissues were embedded in optimal cutting temperature (OCT) compound and sectioned with a freezing microtome at a thickness of 40 μm. These sections were imaged via confocal laser scanning microscopy (Nikon, Japan).

### 2.12 In Vivo Multichannel Electrophysiological Recording

The frequency of neuronal firing in the NTS was recorded. The mice were injected with 250 nL of photogenetic virus (rAAV2/9‐CaMKIIα‐ChR2‐WPRE‐hGH) into the NTS and with chemogenetic virus (rAAV2/9‐CaMKIIα‐hM4D(Gi)‐mCherry‐WPRE‐hGH) into the VPM. Fourteen days later, the mice were anesthetized with 2% isoflurane and then fixed on a brain stereotactic device (RWD, China). An incision was made in the middle of the scalp to expose the skull. The target area was localized, and a drill was used to drill a suitable‐sized cranial window in the skull. Fiber optics were implanted 2.5 mm above the virus injection in the NTS, multichannel electrodes were implanted into S1, and the annulus used for modeling was fixed with dental cement. The mice were then allowed to recover for 7 days. Recordings and further analysis were performed using a Plexon system (OmniPlex, Plexon, USA). Neuronal activity in S1 was recorded via in vivo multichannel recording in the normal, activation of the NTS, and simultaneous activation of the NTS and inhibition of the VPM. After recording, the mice were sacrificed, and brain sections were acquired for confirmation of the injection site of the viruses.

### 2.13 Optogenetic Combined EMG Recording

Photogenetic activation virus systems were stereotactically injected into the NTS and VPM, respectively (rAAV2/1‐CaMKIIα‐Cre‐WPRE‐hGH and rAAV2/9‐CaMKIIα‐Dio‐hChR2‐EGFP‐WPRE‐hGH). 14 days after virus injection, optical fibers (Newton, China) were implanted into S1, and the mice were allowed to recover for 7 days. After recovery, optogenetic activation was performed while the EMG responses of the mylohyoid were recorded. The parameters of optogenetic stimulation were as follows: power of 8 mW and 50 Hz for 5 s. The parameters of the water‐induced swallowing EMG response were as follows: speed, 2 μL/s; duration, 10 s; and interval, 1 min. A minimum of three mouthfuls were collected from each mouse. Brain tissues injected with the virus were collected for IF to confirm the site of virus expression.

### 2.14 Chemical Genetic Inhibition Combined With EMG Recording

The chemogenetic activation virus systems were stereotactically injected into the NTS and VPM (rAAV2/1‐CaMKIIa‐Cre‐WPRE‐hGH and rAAV2/9‐CaMKIIa‐Dio‐hM4Di‐EGFP‐WPRE‐hGH), respectively. 14 days after virus injection, a drug delivery catheter (Newton, China) was implanted into S1 for CNO injection, and the CNO was dissolved in the artificial cerebrospinal fluid (aCFS). After 7 days of recovery, chemogenetic inhibition was performed simultaneously with the recording of the EMG, in the baseline state, aCFS injection and CNO+aCFS injection. aCFS and CNO+aCFS were administered successively, and after 5 min of drug response, EMG recording was performed to observe the changes in the EMG responses. The parameters of the water‐induced EMG response were as follows: speed, 2 μL/s; duration, 10 s; and interval, 1 min. The delivery parameters for S1 were: total drug volume 300 nL, delivery rate 50 nL/s for 6 s. A minimum of three mouthfuls were collected from each mouse. Brain tissues injected with the virus were collected for IF staining to confirm the site of virus expression. Furthermore, to validate the neural circuit mechanism, we employed a dual‐virus strategy:NTS (AAV1‐CaMKIIα‐Cre) and S1(AAVretro‐DIO‐hM4Di‐mCherry). After 21 days of viral expression, we administered either CNO or aCSF control through the implanted VPM cannula to examine how circuit inhibition modulates EA‐CV23's therapeutic effects on swallowing. Experimental procedures were performed as previously described.

### 2.15 Data Analysis and Statistical Tests

For the animal experiments, normally distributed data were analyzed via one‐way ANOVA with Tukey's post hoc test, unpaired *t* test, and variance analysis of random blocks, and non‐normally distributed data were analyzed via the Kruskal–Wallis test with Tukey's post hoc test. Statistics with two tests (*F* values or *p* values) were given in the figure legends. Unpaired Student's *t* tests were used for inter‐group comparisons, Paired *t* tests or Mann Whitney Wilcoxon were used for pre‐ and post‐treatment comparisons of the same subjects with normal distributions. Data analysis was performed by an experimenter blinded to the experimental conditions. All the statistical analyses were performed via SPSS (version 21.0) and GraphPad Prism (version 7.0). The data are presented herein as the means ± standard deviations (SDs), with error bars in the graphs denoting the SDs. Statistical significance was set as *p* < 0.05.

## 3 Results

### 3.1 Gold Standard of Swallowing Function Is Used to Validate a Mouse Model of Dysphagia After Cortical Infarction

Owing to the important role of M1 in the onset of voluntary swallowing [10, 25], we constructed a PSD mouse model via photochemical embolization according to our previous study [10]. Consistent with the previous study, the LSCI results revealed obvious infarct foci in the PSD group, with a significant decrease in the perfusion of M1 when compared with the normal group (*p* < 0.05, Figure 1a,b). To verify the presence of impaired swallowing function in the PSD model, EMG of the mylohyoid, a method used in our previous study to assess the swallowing function of PSD, was applied in this study. The results showed that the AUC of EMG in the PSD group was lower than that in the normal group (*p* < 0.05, Figure 1c,d). However, the gold standard evaluation of dysphagia in clinical practice is VFSS and FEES [22, 24], which directly displays the swallowing‐related structures through imaging, so as to calculate parameters such as swallowing time and compare swallowing function. For this purpose, VFSS and FEES were used to assess and verify the presence of swallowing disorders in the mouse model. The results of VFSS indicated that compared with those in the normal group, the bolus area in the PSD group was significantly smaller (*p* < 0.05, Figure 1f). Representative images are shown in Figure 1e. In addition to the size of the swallowed amount, the swallowing time is also affected. The PTT in the PSD group was significantly prolonged compared with that in the normal group (*p* < 0.05, Figure 1g). However, no difference was observed in the ETT and OTT between the groups (Figure 1g), while the same results were obtained for the total swallow time (Figure 1h). For the results of FEES, the vocal cord activity within 10 s was lower in the PSD group when compared with the normal group (*p* < 0.05, Figure 1i,j). At the same time, the vocal cord motility was prolonged in the PSD group (*p* < 0.05, Figure 1k), but no difference in the mean motion range ratio (MMRR) was observed between the groups (Figure 1l). Furthermore, the FEES showed the residual in the pharyngeal cavity and pyriform crypts of PSD mice (*p* < 0.05, Figure S1a).Additionally, body weight assessment revealed significant weight loss in PSD model mice post‐modeling, while TTC staining and HE staining demonstrated distinct ischemic foci and tissue damage (Figure S9a–c).

### 3.2 EA‐CV23 Improves the Swallowing Function of PSD Model Mice

**FIGURE 2:**
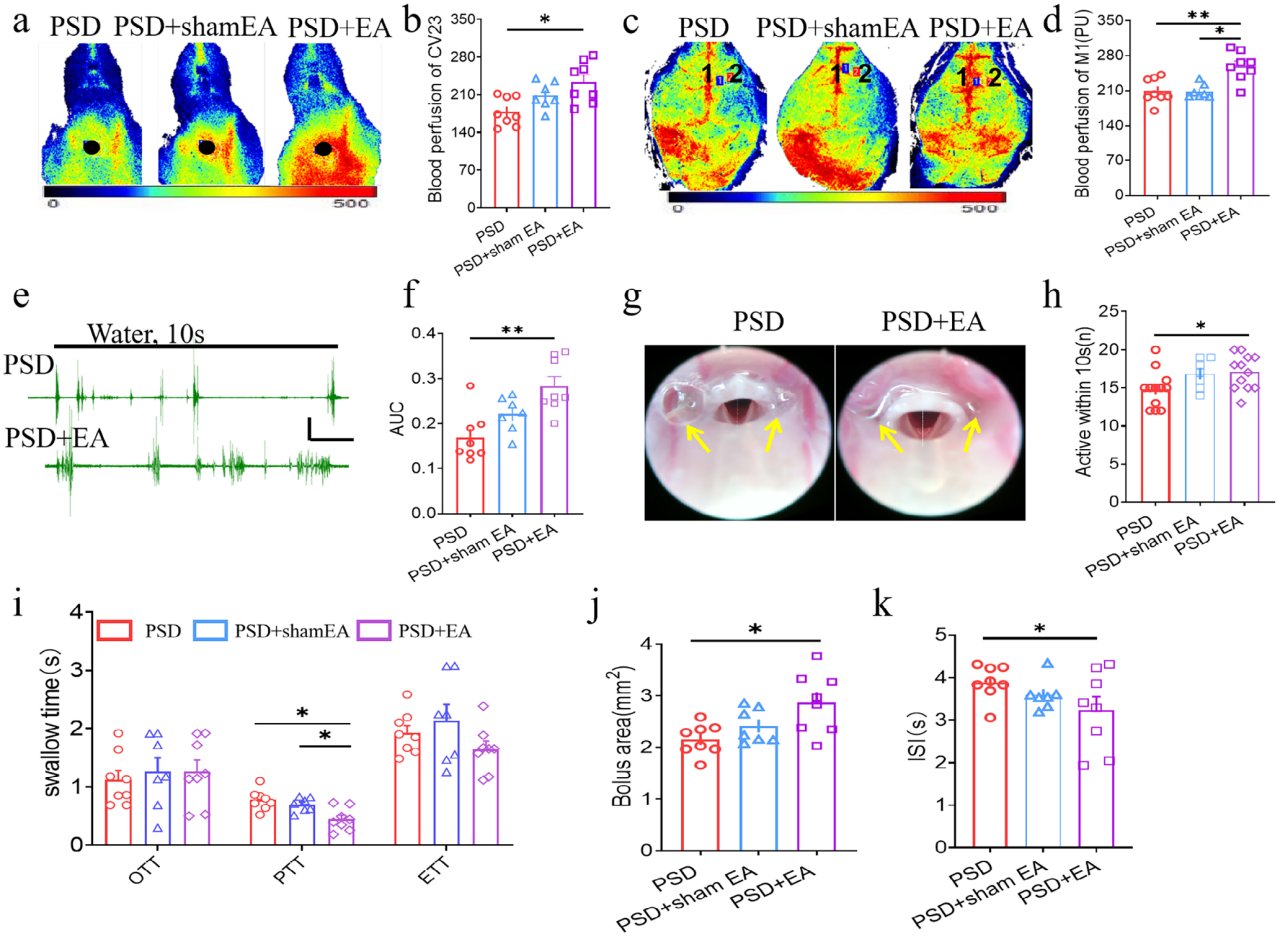
EA at CV23 improved the swallowing function in PSD mouse model. (a) Representative example of LSCI showed the blood perfusion of the CV23 in the PSD, PSD + sham EA, and the PSD + EA groups. Duration: 120 s, Target area = 4 mm^2^. (b) EA increased the blood perfusion of CV23 in the PSD + EA mice (One‐way ANOVA with Bonferroni posthoc test, *F* = 2.609, *p* = 0.086, PSD, *n* = 8, PSD + sham EA, *n* = 7, PSD + EA, *n* = 8; *p < 0.05, PSD vs. PSD + EA). (c) Representative example of the blood perfusion of M1 and S1 in the PSD, PSD + sham EA, and the PSD + EA groups. Duration: 120 s, target area = 4 mm^2^, target 1 (black): M1, target 2 (red): S1. (d) EA increased the blood perfusion of M1 in the PSD + EA mice (One‐way ANOVA with Bonferroni posthoc test, *F* = 10.285, *p* = 0.001 PSD, *n* = 8, PSD + sham EA, *n* = 7, PSD + EA, *n* = 8; **p < 0.001, PSD vs. PSD + EA,*p < 0.05, PSD+ sham EA vs. PSD + EA). (e) A representative example of EMG in the PSD and the PSD + EA groups. Black line: Water administration for 10 s with a velocity of 2 μL/s. Scale: 0.2 mV, 1 s. (*f*) EA increased the AUC of EMG in the PSD + EA group (One‐way ANOVA with Bonferroni posthoc test, *F* = 9.535, *p* = 0.001, PSD, *n* = 8, PSD + sham EA, *n* = 7, PSD + EA, *n* = 8; *p < 0.001, PSD vs. PSD+ EA). (g) Representative example of FEES showed the secretion residues in PSD and PSD + EA group. Yellow arrow: Secretion. (h) EA increased the number of vocal fold activities over 10 s in the PSD + EA group (One‐way ANOVA with Bonferroni posthoc test, *F* = 3.345. *p* = 0.050, PSD, *n* = 8, PSD + sham EA, *n* = 7, PSD + EA, *n* = 8; *p = 0.022, PSD vs. PSD+ EA). (i) EA shortened PTT in the PSD + EA group (One‐way ANOVA with Bonferroni posthoc test, *F* = 8.556, *p* = 0.002, PSD, *n* = 8, PSD + sham EA, *n* = 7, PSD + EA, *n* = 8; *p* = 0.009, PSD + sham EA vs. PSD + EA;*p = 0.001, PSD vs. PSD+ EA). (j) The bolus area was significantly higher in the PSD + EA group than that in the PSD group (One‐way ANOVA with Bonferroni posthoc test, *F* = 5.570, *p* = 0.012, PSD, *n* = 8, PSD + sham EA, *n* = 7, PSD + EA, *n* = 8; *p = 0.004, PSD vs. PSD+ EA). (k) EA reduced ISI in the PSD + EA group (One‐way ANOVA with Bonferroni posthoc test, *F* = 2.230, *p* = 0.134 PSD, *n* = 8, PSD + sham EA, *n* = 7, PSD + EA, *n* = 8; *p = 0.048, PSD vs. PSD+ EA).

The effect of EA treatment for dysphagia had been verified in our previous study with the EMG and water consumption [10]. Here, we further validate the therapeutic effect of EA by using the gold standard for the evaluation of dysphagia. Consistent with previous studies, the results revealed that blood perfusion in the localization of CV23 and M1 was significantly greater in the EA group than in the PSD group (*p < 0.05, Figure 2a–d). So was the S1, which indicated that EA might strengthen the blood supply of the sensory cortex (*p < 0.05, Figure S1b). Similar results were seen in the AUC of EMG of mylohyoid (*p < 0.05, Figure 2e,f). In terms of the pharyngeal structure, the results of FEES revealed that the number of activities in 10 s for vocal fold was significantly greater in the EA group compared with the model group (*p* < 0.05, Figure 2g,h), but there was no difference in vocal fold motion (*p* > 0.05, Figure S1c). A large amount of residue was found in the pharyngeal cavity of the PSD group (Figure S1a). The VFSS results further revealed that, compared with the PSD group, the PTT in the EA group was significantly reduced (*p < 0.05. Figure 2i).However, the differences in the time of inter swallowing intervals (ISI) were not statistically significant (Figure 2k). And the bolus area increased after EA treatment in the EA group compared with the PSD group (*p < 0.05. Figure 2i). VFSS images show the swallowing process between the two groups (Figure S1d). Combining the results of blood perfusion, EMG, imaging, and structure detection, we concluded that EA can improve the swallowing function of PSD model mice.

### 3.3 EA‐CV23 Can Activate the Excitatory Neurons in VPM

**FIGURE 3:**
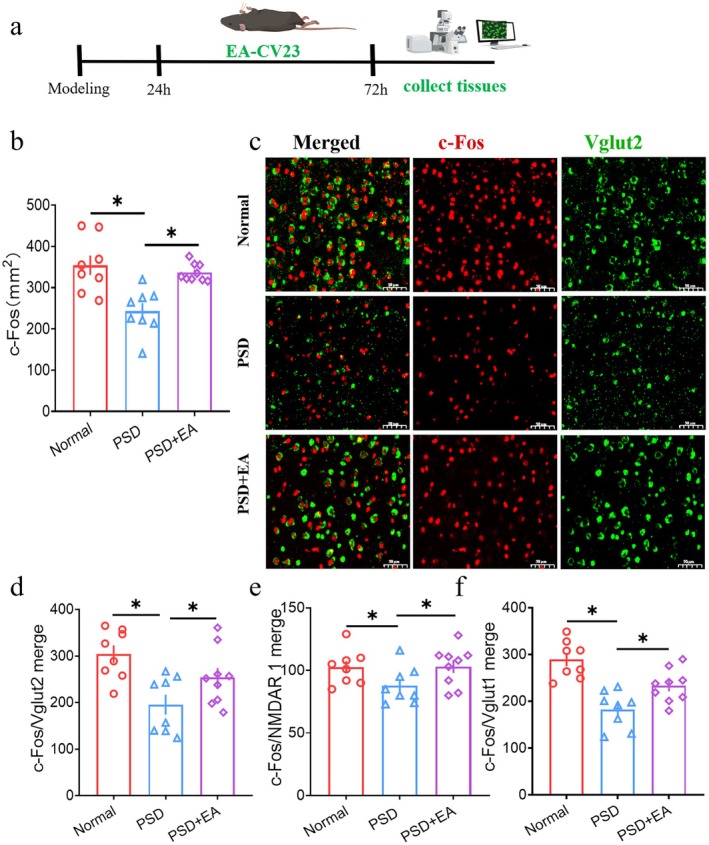
Excitaory neurons in VPM were activated by EA‐CV23. (a) Schematic illustration of the experimental design. (b) Expression of c‐Fos was lower in the PSD group than in the normal and PSD + EA groups (One‐way ANOVA with Bonferroni posthoc test, *F* = 14.500, normal group: *N* = 8, PSD group: *N* = 8, EA group: *N* = 9; *p < 0.05, PSD vs. normal, PSD vs. PSD + EA). (c) Representative example of the coexpression of c‐Fos and Vglut2 of VPM in the three groups. Scale bar, 50 μm. (d) Co‐expression of c‐Fos and Vglut2 neurons was lower in the PSD group than in the PSD + EA group (One‐way ANOVA with Bonferroni posthoc test, *F* = 14.474, normal group: *N* = 8, PSD group: *N* = 8, PSD + EA group: *N* = 9; *p < 0.05, PSD vs. normal, PSD vs. PSD + EA). (e) Co‐expression of c‐Fos and NMDAR1 neurons was lower in the PSD group than in the PSD + EA group. Scale bar, 50 μm (One‐way ANOVA with Bonferroni posthoc test, *F* = 5.175, normal, *n* = 6, PSD, *n* = 8, PSD + EA, *n* = 9; *p < 0.05, PSD vs. normal, PSD vs. PSD + EA). (f) Co‐expression of c‐Fos and Vglut1 neurons was lower in the PSD group than in the PSD + EA group (One‐way ANOVA with Bonferroni posthoc test, *F* = 10.259, normal, *n* = 8, PSD, *n* = 8, PSD + EA, *n* = 9; *p < 0.05, PSD vs. normal, PSD vs. PSD + EA). Scale bar, 50 μm.

In addition to the well‐known cortex and brainstem involved in normal swallowing, subcortical areas including the thalamus also play an important key role [28]. According to the relevant data, VPM was activated during swallowing activity and may act as a relay station for the transmission of swallowing sensory information, which was thought to be the subcortical sensory integration center during swallowing [29, 30]. To observe the function of VPM during EA‐CV23 treatment for dysphagia, neurons activated after EA in the VPM were first detected by IF. The results revealed that the expression of c‐Fos positive neurons in the PSD group was lower than that in the normal group, which was reversed by EA treatment (*p* < 0.05, Figure 3a,b, Figure S2a). On the basis of the previous studies in which excitatory neurons were activated and acted as an important role in the process of EA‐CV23 for PSD [10, 26], the co‐expression of glutamate neurons like Vglut2, Vglut1, and NMDAR1 with c‐Fos were detected (Figure 3c, Figure S2b,c). The co‐expression of c‐Fos and Vglut2 was significantly greater in the PSD + EA group than that in the PSD group (*p <* 0.05, Figure 3c,d). The results for Vglut1 and NMDAR1 were consistent with those for Vglut2 (*p <* 0.05, Figure 3e,f, Figure S2b,c). These findings further indicated that excitatory neurons in the VPM could be activated by EA‐CV23 in the PSD mouse model.

### 3.4 VPM is involved in the EA‐CV23–induced improvement of swallowing function in the PSD mice

**FIGURE 4:**
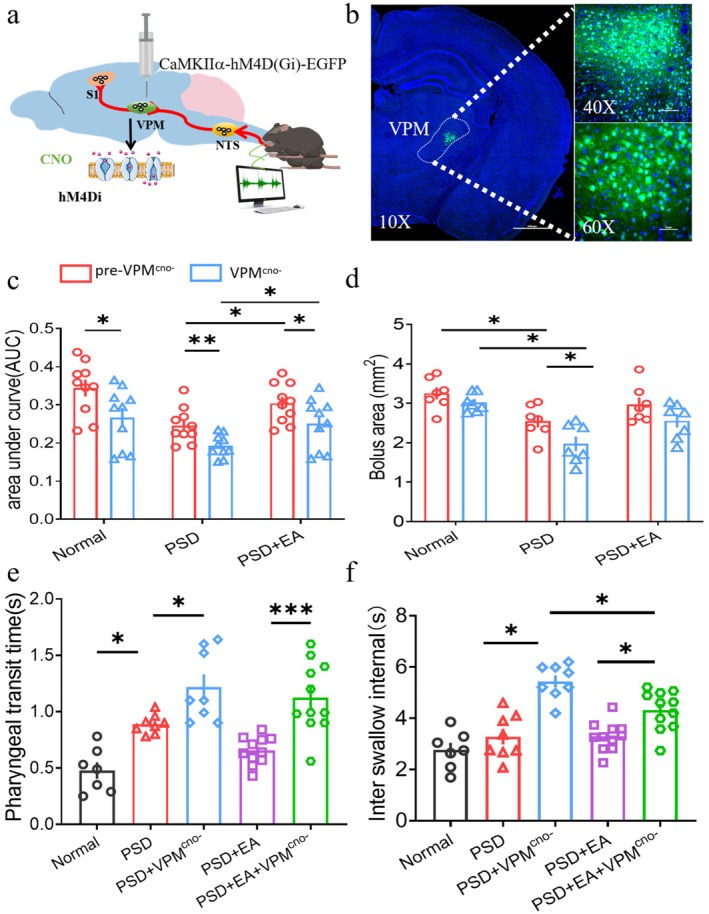
VPM was involved in the EA‐CV23 induced improvement of swallowing function in the PSD mice. (a) Experimental diagram of selective inhibition of VPM. A chemogenetic virus AAV2/9‐CaMKIIα‐hM4Di was injected into VPM. After 21 days of expression, CNO was intraperitoneally injected to inhibit the excitatory neurons of VPM, and EMG was recorded simultaneously. (b) Expression of hM4Di virus in the VPM. The white dotted line marks the VPM area, green: HM4Di virus, blue: DAPI. Scale bar, left: 500 ∝μ, top: right 100 ∝μ, bottom right: 50 ∝μ. (c) AUC in all three groups decreased after inhibiting VPM (VPM^cno−^); this effect was restored by EA in the PSD + EA group. (Two‐tailed Student's paired *t* test, *n* = 10, compare pre‐VPM^cno−^ with VPM^cno−^ within groups: Normal group: *T* = −5.224,*p < 0.05, PSD group: *T* = −5.224, **p < 0.01, PSD + EA group: T = 2.821, *p < 0.05; One‐way ANOVA with Bonferroni posthoc test, *F* = 4.046, *n* = 10 per group, comparison between groups in pre‐VPM^cno−^ *p < 0.05, PSD vs. PSD + EA; comparison bteween groups in post‐VPM^cno−^, *p < 0.05; PSD + VPM^CNO−^ vs. PSD + EA + VPM^CNO^,0.19 ± 0.029 vs. 0.25 ± 0.663). (d) Inhibition of VPM decreased the bolus area in the PSD group after VPM^CNO−^ (Two‐tailed Student's paired *t* test, *t* = 2.562, *n* = 7, *p < 0.05). The bolus area both decreased in the pre‐VPM^cno− CNO−^ and VPM in the PSD group compared to the normal group (One‐way ANOVA with Bonferroni posthoc test, *F* = 1.767, normal group: *N* = 7; PSD group; *n* = 7; EA group: *N* = 11, pre‐VPM^cno−^ *p < 0.05, PSD vs. normal*;* VPM^CNO−^: **p < 0.01, PSD vs. normal. However, with no significantly difference between PSD and PSD + EA groups). (e) Inhibition of VPM significantly lengthened the PTT in both the PSD group and PSD + EA group (PSD group; *n* = 8; EA group: *N* = 11. Mann Whitney Wilcoxon, Z = −2.521,*p < 0.05, PSD vs. PSD + VPM^cno−^; Z = −2.845, ***p < 0.001, PSD + EA vs. PSD + EA + VPM^cno−^). (f) Inhibition of VPM significantly lengthened the ISI in the PSD group and the PSD + EA group (normal group: *N* = 7; PSD group; *n* = 7; EA group: *N* = 11. Mann Whitney Wilcoxon, Z = −2.521,*p < 0.05, PSD vs. PSD + VPM^cno−^; Mann Whitney Wilcoxon, Z = −2.756, *p < 0.05, PSD + EA vs. PSD + EA + VPM^cno−^; Kruskal–Wallis test, *F* = 14.453,*p < 0.05, PSD + VPM^cno−^ vs. PSD + EA + VPM^cno−^).

We subsequently explored the effects of the VPM on swallowing function in the PSD mice. A chemogenetic inhibition virus AAV2/9‐CaMKIIα‐hM4Di‐EGFP was injected into the VPM to inhibit the excitatory neurons, and the EMG was detected (Figure 4a,b). Results demonstrated that inhibition of the VPM significantly reduced the EMG when compared with the pre‐inhibition, no matter in the normal, PSD, and PSD + EA group (*p <* 0.05, Figure 4c, Figure S3a–c). Before inhibition, EA‐CV23 increased the EMG response of PSD mice in the PSD + EA group compared with the PSD group, suggesting that EA‐CV23 could improve the swallowing function of PSD mice. The same effect still existed after intraperitoneal injection of CNO for the inhibition of VPM (*p <* 0.05, Figure 4c).

To further verify the role of VPM on swallowing function of PSD mice, the swallowing performance at different swallowing phases was detected via the VFSS. Consistent with the results of previous studies, the bolus area in the PSD group was reduced compared with the normal group. This effect still existed after inhibition of VPM, which indicated VPM played an important role in the swallowing function of PSD mice. However, only an increasing trend was seen in the PSD + EA group compared with the PSD group after VPM inhibition, with no significant difference (*p >* 0.05, Figure 4d). Comparing the total swallowing time before and after inhibition of VPM, only PTT was found to have a significant difference among the three groups (*p <* 0.05, Figure 3a–c). PTT was then analyzed separately. Compared with the normal group, PTT in the model group was significantly increased (*p <* 0.05, Figure 4e). Inhibition of VPM prolonged PTT in the PSD + VPM^cno−^ group when compared with the PSD group (*p <* 0.05, Figure 4e), and the same result was found in the PSD + EA + VPM^cno−^ group and PSD + EA group (*p <* 0.001, Figure 4e). But no significant difference was seen between the PSD + VPM^cno−^ group and PSD + EA + VPM^cno−^ group (*p >* 0.05, Figure 4e). However, the inter swallow interval (ISI) increased after VPM inhibition whether in the PSD or PSD + EA group (*p <* 0.05, Figure 4f). Even more, ISI in the PSD + EA + VPM^cno−^ group was lower than that in the PSD + VPM^cno−^ group, suggesting that the effect of inhibiting VPM to increase the swallowing interval could be improved by EA‐CV23 (*p <* 0.05, Figure 4f). These findings suggest that the VPM is involved in and may even play a key role in improving swallowing function in PSD model mice via EA‐CV23.

### 3.5 Direct Excitatory Synaptic Connections Exist in the NTS‐VPM‐S1 Neural Circuit

**FIGURE 5:**
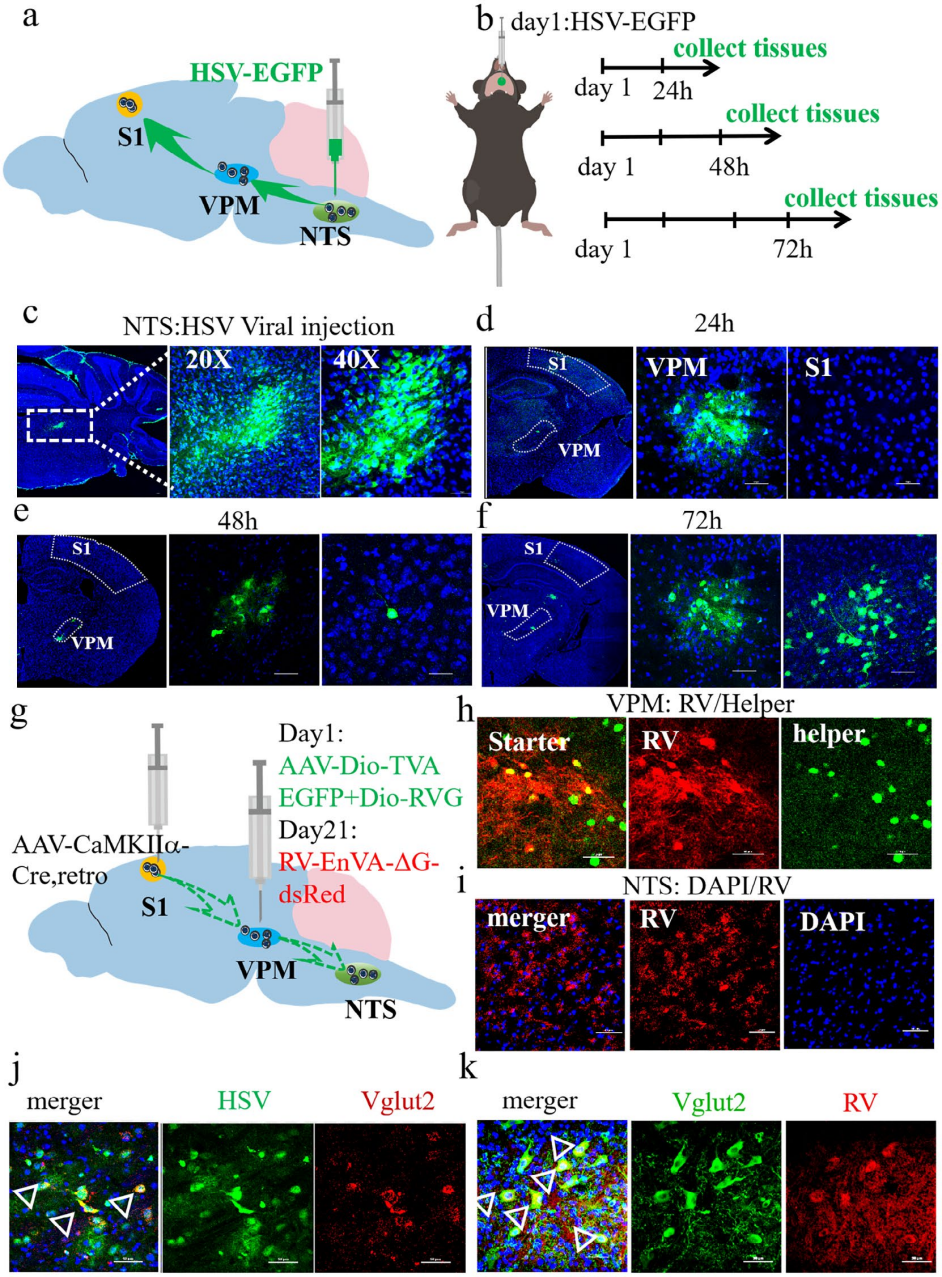
Direct excitatory synaptic connections exist in the NTS‐VPM‐S1 neural circuit. (a) Schematic diagram of the HSV virus injection. (b) Schematic illustration of the virus tracing experimental design. The virus was injected on the first day and tissues of VPM and S1 were collected in the following 24, 48, and 72 h respectively. (c) Representative diagram of HSV expression in NTS, Left: The HSV virus (green) was detected in NTS, scale bar, 500um. Intermediate: The enlarged versions of the images on the left. Scale bar, 100um. Right: The enlarged versions of the images on the left. Scale bar, 50 μm. (d) A representative diagram of transfected neurons in VPM 24 h after injection of HSV virus in NTS, but not S1. Scale bar, 50 μm. (e) Representative diagram of a large number of positively transfected neurons in VPM and a little of transfected neurons in S1 48 h after HSV virus injection. Scale bar, 50 μm. (f) Representative diagram of considerable positively transfected neurons in both VPM and S1 72 h after HSV virus injection. Scale bar, 50 μm. (g) Schematic illustration of the retrograde tracer experimental design with AAV2/9‐CaMKIIα‐Cre‐retro injected into S1 and helper virus (AAV2/9‐Dio‐TVA and AAV2/9‐Dio‐RVG) injected into VPM simultaneously, following with the RV injected into VPM again 14 days later. (h) Representative diagram of starter neurons expressed in the VPM. red: RV virus, green: Helper virus. Scale bar, 50 μm. (i) Representative diagram of retrograde projection neurons expressed in the NTS. Scale bar, 50 μm. (j) Representative diagram of the co‐expression of HSV neurons (green) projected from the NTS to VPM with Vglut2 (red). Scale bar, 50 μm. (k) Representative diagram of the co‐expression of RV neurons (red) with Vglut2 (green) in NTS. Scale bar, 50 μm.

NTS is the primary sensory afferent nucleus of swallowing, and its excitatory neurons may play a key role in the treatment of PSD by EA‐CV23 [27]. The S1, the higher center for processing pharyngeal sensations, is activated during swallowing [26]. To investigate whether the VPM is involved in the swallowing process as a relay station of NTS‐S1, neural tracing was used. First, an anterograde polysynaptic virus HSV‐EGFP was injected into the NTS, and synaptic projections were detected in the VPM and S1 at 24 h, 48 h, and 72 h, respectively (Figure 5a–f). 24 h after virus injection, a small number of transfected EGFP‐labeled neurons were detected in the VPM but none in S1 (Figure 5d). After 48 h, a greater number of transfected neurons were observed in the VPM, and fewer transfected neurons were observed in S1 (Figure 5e). After 72 h, there were a significantly considerable number of transfected neurons in both VPM and S1 (Figure 5f). This suggests that the VPM may act as a relay station from NTS to S1 and that the synaptic projection of the NTS first passes through the VPM and then reaches S1.

To further verify the direct synaptic connections between the NTS, VPM, and S1, monosynaptic retrograde RV virus was used, with AAV‐CaMKIIα‐Cre‐retro injected into S1 and helper viruses (AAV2/9‐Dio‐RVG and AAV2/9‐Dio‐EGFP‐TVA) injected into the VPM simultaneously. 14 days later, RV‐ΔG‐dsRed was injected into the VPM again, and the expression of neurons in the NTS was detected 7 days later (Figure 5g). A considerable number of initiating neurons were detected in the VPM (Figure 5h, Figure S6a,b), and red‐positive neurons were observed in the NTS (Figure 5i), suggesting that VPM neurons receive retrograde inputs from S1 and further retrograde projections to the NTS. Therefore, the presence of the direct NTS‐VPM‐S1 neural circuit can be confirmed.

Based on the previous findings of this study, the colocalization of Vglut1, Vglut2, and NMDAR1 neurons with anterograde projection neurons in the S1 and VPM was performed. The results revealed that the colocalization of HSV‐transfected neurons in the VPM with Vglut2 and NMDAR1 neurons significantly overlapped, but only a small number of Vglut1 neurons were colocalized (Figure 5j, Figure S4a,b). Nevertheless, RV‐transfected NTS neurons were significantly colocalized only with Vglut2 (Figure 5k), while no colocalization of NMDAR1 and Vglut1 with RV‐transfected neurons was found (Figure 4c,d). The above results suggested that the type of synaptic connection of the NTS‐VPM‐S1 neural circuit was excitatory synapses.

### 3.6 Neuronal Excitability of S1 Can Be Activated by EA‐CV23 and Participates in the NTS‐VPM Neural Circuit

**FIGURE 6:**
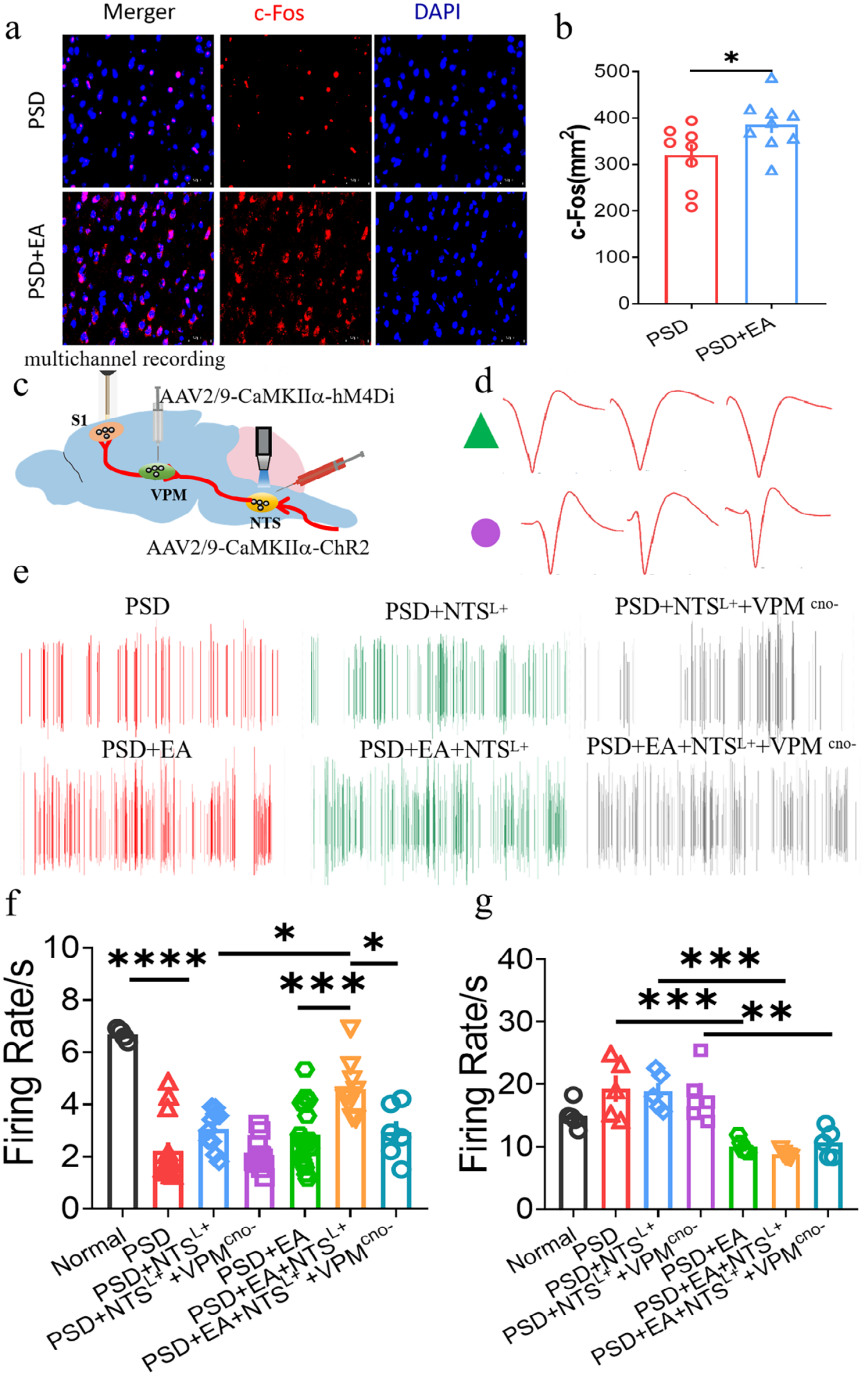
Neuronal excitability of S1 can be activated by EA‐CV23 and participates in the NTS‐VPM neural circuit. (a) Representative immunofluorescence images showing c‐Fos (red) and DAPI (blue) co‐expression in S1 cortex of PSD and PSD + EA groups. Scale bar = 50 μm. (b) EA activated c‐Fos positive neurons of S1 in the PSD mice (Two‐tailed Student's unpaired *t* test, *t* = −2.24, PSD, *n* = 8, PSD + EA, *n* = 9; *p < 0.05, PSD vs. PSD + EA). (c) Diagram showing optogenetic activation of NTS neurons and inhibition of VPM neurons. AAV2/9‐CaMKIIα‐ChR2‐EGFP was injected into the NTS with light applied, while AAV2/9‐CaMKIIα‐hM4Di‐mCherry was injected into the VPM with CNO intraperitoneally injected, following with in vivo multichannel electrophysiologic recording for S1. (d) Representative figure of interneurons and pyramidal neurons. Green, pyramidal neuron. Purple, interneuron. (e) The representative firing figure of S1 neurons in PSD and PSD + EA groups. Time:120 s. (f) After EA treatment, the firing rate of S1 pyramidal neurons increased after activation of NTS, while decreased significantly after inhibition of VPM simultaneously (One‐way ANOVA with Bonferroni posthoc test, PSD, *n* = 5, PSD + EA, *n* = 5, ****p < 0.0001, normal vs. PSD; *p < 0.05, PSD + NTS^L+^ vs. PSD + EA + NTS^L+^, PSD + EA + NTS^L+^ vs. PSD + EA + NTS^L+^+VPM^cno−^; ***p < 0.001, PSD + EA vs. PSD + EA + NTS^L+^). (g) EA could increase the activity of interneurons in the S1, and this response was also present in activation of NTS, and simultaneously activation of NTS and inhibition of VPM (One‐way ANOVA with Bonferroni posthoc test, PSD, *n* = 5, PSD + EA, *n* = 5, ***p < 0.001, PSD vs. PSD + EA, PSD + NTS^L+^ vs. PSD + EA + NTS^L+^; **p < 0.01, PSD + NTS^L+^+VPM^cno−^ vs. PSD + EA + NTS^L+^+VPM^cno−^).

Circuit tracing revealed polysynaptic connectivity between S1 and NTS via VPM relay (Figure 5). To further observe the role of S1 in EA treatment for PSD mice, we first observed the neuronal activity of S1 via IF after EA‐CV23. Results showed that EA could increase the c‐Fos positive neurons of S1 in the PSD + EA group when compared with the PSD group (Figure 6a,b, Figure S7). Next, to explore whether the neuronal activity of S1 was regulated by the NTS and VPM, AAV virus carrying an excitatory starter CaMKIIα (AAV2/9‐CaMKIIα‐ChR2 and AAV2/9‐CaMKIIα‐hM4Di) was injected into NTS and VPM respectively to achieve the optogenetic activation of the NTS and chemogenetic inhibition of the VPM, followed by the recording of neuronal firing via electrodes implanted into S1 (Figure 6c, Figure S5a,b). The recorded neurons were divided into pyramidal neurons and interneurons based on the waveform and firing frequency [31] (Figure 6d,e). For pyramidal neurons, the firing rate of the PSD group was lower than the normal group (*p* < 0.0001, Figure 6f). But no difference was found in the PSD + NTS^L+^ and PSD + NTS^L+^+VPM^cno−^ groups. However, after EA‐CV23, photogenetic activation of NTS significantly increased the neuronal activity of S1 in the PSD + EA + NTS^L+^ group when compared with the PSD + EA group (*p* < 0.001, Figure 6f), and the effect was significantly decreased in the PSD + EA + NTS^L+^+VPM^cno−^ group after chemogenetic suppression of VPM (*p* < 0.05, Figure 6f). Additionally, EA‐CV23 also increased the firing rate of S1 after photogenetic activation of NTS in the PSD + EA + NTS^L+^ group compared with the PSD + NTS^L+^ group, but no significant difference was seen after both photogenetic activation of NTS and chemogenetic inhibition of VPM in the PSD + EA + NTS^L+^+VPM^cno−^ when compared with the PSD + NTS^L+^+VPM^cno−^.

For interneurons, EA‐CV23 could reduce the firing rate of S1 in the PSD + EA group when compared with the PSD group. Similarly, EA‐CV23 decreased the firing rate of S1 interneurons after photogenetic activation of NTS in the PSD + EA + NTS^L+^ group compared with the PSD + NTS^L+^ group. Furthermore, the neurons firing rate of S1 was significantly reduced in the PSD + EA + NTS^L+^ +VPM^cno−^ group when compared with the PSD + NTS^L+^ +VPM^cno−^ group (Figure 6g). On the basis of the above results, we preliminarily concluded that both NTS and VPM had a regulatory effect on S1 neurons, which may play a role in EA treatment for PSD, but more evidence is needed.

### 3.7 The NTS‐VPM‐S1 Neural Circuit Mediates the Protective Effect of EA‐CV23 Against PSD

**FIGURE 7:**
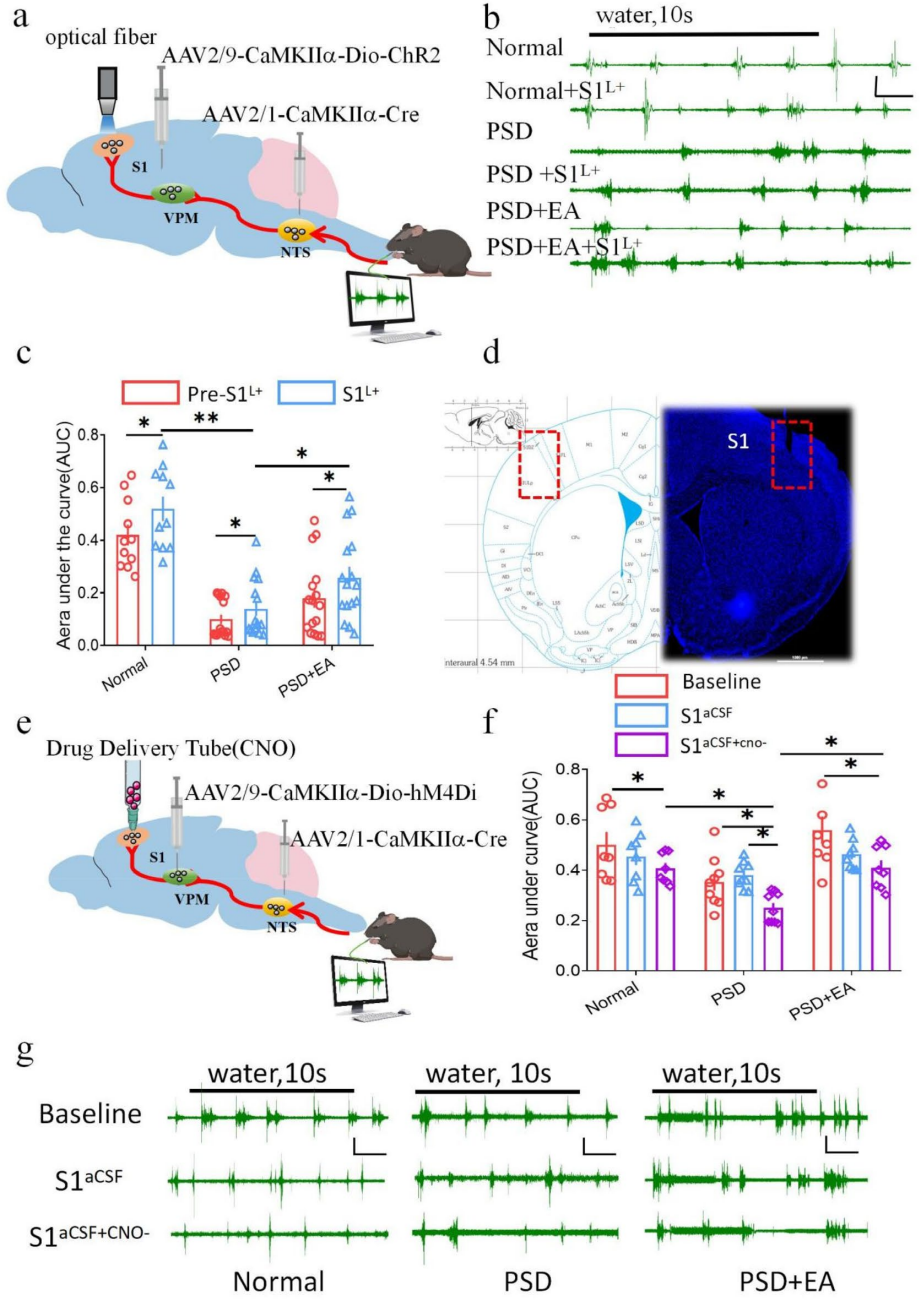
The NTS‐VPM‐S1 neural circuit is involved in the regulation of swallowing function and mediates the protective effect of EA‐CV23 against PSD. (a) Schematic diagram showing selective activation of the NTS‐VPM‐S1 neural circuit. AAV2/9‐CaMKIIα‐Cre was injected into NTS and AAV2/9‐CaMKIIα‐Dio‐ChR2 was injected into VPM respectively, and blue light was delivered to the S1 via an implanted fiber. (b) Representative diagram of EMG in different groups of mice after direct activation of the NTS‐VPM‐S1 neural circuit. Black line: Water administration for 10 s with a velocity of 2 μL/s. Scale: 0.2 mV, 1 s. (c) After activation of the NTS‐VPM‐S1 neural circuit, EA enhances the EMG responses (PSD + S1^L+^ vs. PSD + EA + S1^L+^) (one‐way ANOVA, *N* = 12 per group; *F* = 24.61; post hoc Tukey's test: **p < 0.01, normal+S1^L+^ vs. PSD + S1^L+^, *p < 0.05, normal+Pre‐S1^L+^ vs. normal+S1^L+^, PSD + Pre‐S1^L+^ vs. PSD + S1^L+^, PSD + EA + Pre‐S1^L+^ vs. PSD + EA + S1^L+^, PSD + S1^L+^ vs. PSD + EA + S1^L+^). (d) Representative diagram of fiber optic implanted in S1. Fiber optic traces are shown in the red box. (e) Schematic diagram showing selective inhibition of the NTS‐VPM‐S1 neural circuit. AAV2/1‐CaMKIIα‐Cre was injected into NTS and AAV2/9‐CaMKIIα‐Dio‐hM4Di was injected into VPM respectively, followed by CNO or artificial cerebrospinal fluid (CSF) delivered to the S1 via an implanted tube. (f) The AUC of EMG responses was decreased after inhibition of the neural circuit, and EA enhances swallowing (PSD + S1^aCSF+cno−^ vs. PSD + EA + S1^aCSF+cno−^) (one‐way ANOVA, *n* = 12 per group; *F* = 15.178; post hoc Tukey's test: *p < 0.05). (g) Representative diagram of EMG in different groups of mice after activation of the NTS‐VPM‐S1 neural circuit. Black line: Water administration for 10 s with a velocity of 2 μL/s. Scale: 0.2 mV, 1 s.

To further determine whether these brain regions collectively regulate the swallowing function of the PSD model, we regulated the direct neural circuit of the NTS‐VPM‐S1. First, optogenetics combined with EMG recording was used to regulate this direct neural circuit. AAV2/1‐CaMKIIα‐Cre was injected into the NTS, and AAV2/9‐CaMKIIα‐Dio‐ChR2 was injected into the VPM, along with an optical fiber implanted into S1 to selectively activate NTS‐innervated VPM excitatory neurons projecting to the end of S1, followed by water‐induced EMG recording to identify the role of the NTS‐VPM‐S1 neural circuit in EA‐CV23 improvement of swallowing function (Figure 7a,b). The results showed that activation of the NTS‐VPM‐S1 neural circuit (S1^L+^) resulted in a significant increase in AUC compared with the Pre‐S1^L+^ conditions in the three groups (*p* < 0.05, Figure 7c). Interestingly, after activating the NTS‐VPM‐S1 neural circuit, the EMG response in the PSD group was lower than that in the PSD + EA group, and the difference was statistically significant (*p* < 0.01, Figure 7c). Furthermore, compared with the model group, EA could further increase the EMG response after activating this circuit (*p* < 0.01, Figure 7c).These results suggested that optogenetic activation of the NTS‐VPM‐S1 neural circuit significantly increased water‐induced EMG responses and that this effect was more significant after EA‐CV23.

Furthermore, to reinforce the above evidence, chemogenetics was conducted with AAV2/1‐CaMKIIα‐Cre injected into the NTS and AAV2/9‐CaMKIIα‐Dio‐hM4Di injected into the VPM, followed by CNO administration into S1 with an cannula to selectively inhibit the NTS‐VPM‐S1 neural circuit (Figure 7e). The results indicated that the EMG response was significantly attenuated after CNO injection, and the EA‐CV23‐mediated improvement in the EMG response was also prevented (*p* < 0.05, Figure 7f,g). After chemogenetic inhibition of NTS‐VPM‐S1 neural circuit (S1^aCSF+cno−^), the EMG response decreased significantly, and the difference was statistically significant compared with baseline. After inhibition of this neural circuit, the EMG in the PSD group (PSD + S1^aCSF+cno−^) decreased significantly compared with the normal group, and EA‐CV23 (PSD + EA + S1^aCSF+cno−^) could salvage this response (*p* < 0.05, Figure 7f,g). To validate the impact of retrograde inhibition of this circuit on EA‐CV23's therapeutic efficacy, our results demonstrate that inhibition of the VPM (via CNO administration) significantly reduced the AUC in all experimental groups. Notably, at baseline and under ACSF conditions, PSD + EA and PSD groups showed significant differences (*p < 0.05), whereas after silencing the NTS‐VPM‐S1 circuit, this distinction was abolished (*p > 0.05). These findings suggest that EA‐CV23's therapeutic effects rely on the NTS‐VPM‐S1 pathway, as its inhibition attenuates EA's efficacy. This provides functional validation of our proposed mechanism. The new data (Figure S8) demonstrate that silencing this circuit significantly attenuates EA‐CV23 therapeutic effects, supporting our original hypothesis.

**FIGURE 8:**
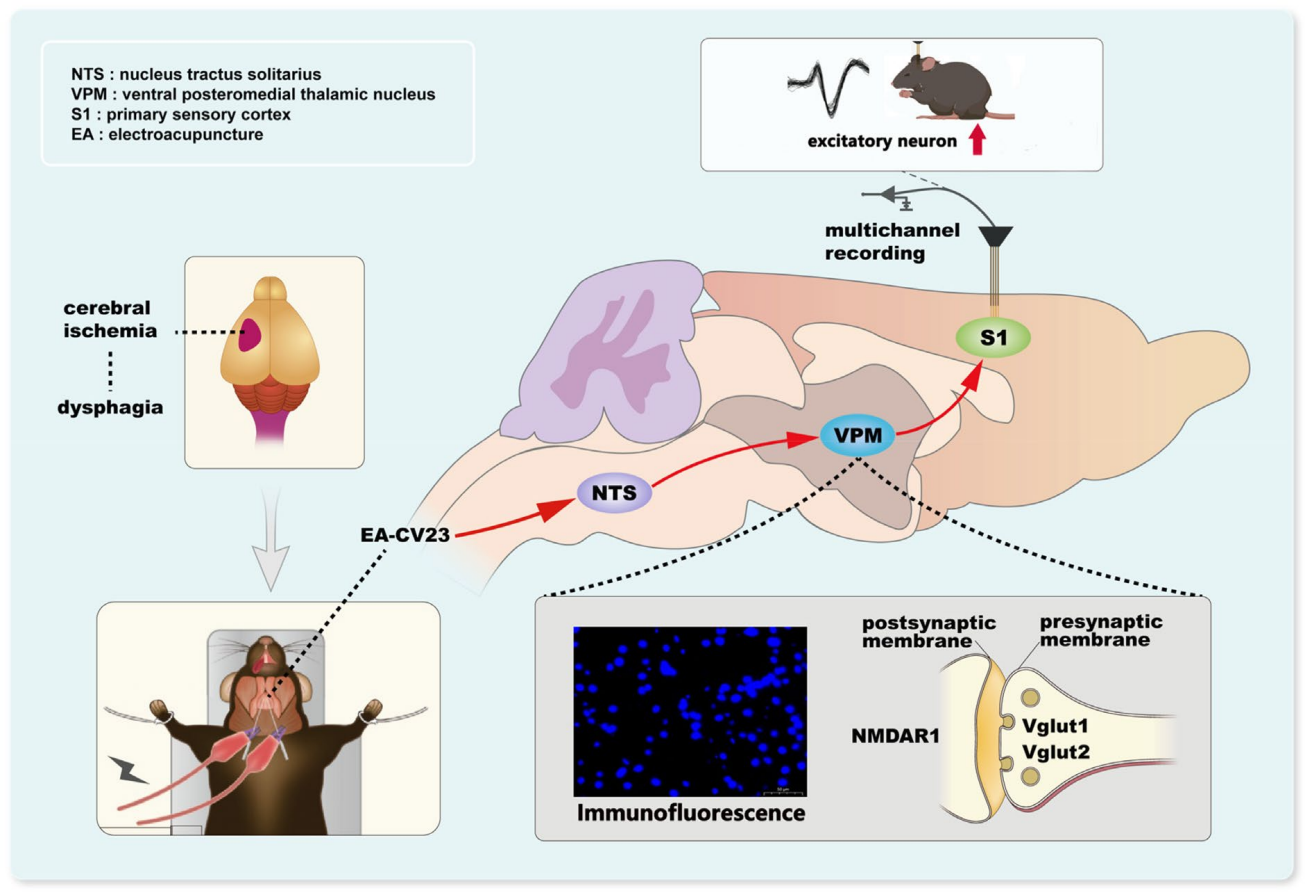
Diagram of the mechanism underlying the effect of EA‐CV23 treatment on PSD.

Taking the above results into account, we concluded that the NTS‐VPM‐NTS neural circuit regulated swallowing function and that these regulatory effects participated in the therapeutic effect of EA‐CV23 on PSD (Figure 8).

Excitatory neurons in the VPM are required for EA‐CV23 mediated alleviation of swallowing dysfunction in PSD model mice. This modulatory effect of EA involves the NTS‐VPM‐S1 neural circuit.

## 4 Discussion

Sensory input is important for promoting the coordination of swallowing [32, 33]. This study is one of the few to report that EA‐CV23 improves the swallowing function of the PSD through the mechanism of sensory afferents. Our study revealed that EA‐CV23 was effective in improving PSD and that the synaptic plasticity of the VPM played an important role. A direct synaptic connection in NTS‐VPM‐S1 was shown to be an important sensory afferent neural circuit that was involved in the modulation of swallowing function by EA‐CV23 in PSD mice. Overall, these results provided valuable insights into the afference of peripheral sensory stimuli to understand how these subcortical and cortical brains interact and their role in sensory afference such as acupuncture, and why the CV23 acupoint can be selected as a suitable peripheral site for PSD treatment in clinical practice.

### 4.1 Current Status of VFSS as a Swallowing Function Evaluation Method for Improving PSD via EA

Over the past 30 years, the VFSS has been considered the gold standard for evaluating swallowing function, but it is used less frequently in current studies of mouse disease models [34]. In these studies, the assessment of swallowing function relied mainly on simple metrics, such as water intake, body mass, and EMG, which lacked quantitative indices compared with those used in clinical assessments; thus, the conclusions may lack credibility [35]. In our study, the VFSS and FEES were used as measurable approaches, which could provide direct, credible clinical evidence [22, 23] and provide a more comprehensive approach to studying animal models of dysphagia. Unlike the studies of Best and Lever et al. [36], a customized small animal VFSS system was applied in our study. The results showed that the PSD model induced by M1 cortical ischemia mainly exhibited dysphagia in the pharyngeal phase, such as prolongation of the swallowing time and changes in the bolus area in terms of mouthfuls, which were characterized by pharyngeal phase dysfunction (Figure 1g). However, the changes in other swallowing phases were not obvious, which suggested that the pharyngeal phase may be an important target for the PSD model associated with M1, and activation of M1 has been shown to be related to pharyngeal phase regulation [37, 38]. Previous studies did not address specific phases of swallowing, and this was an advantage of using a VFSS to assess swallowing function in this study [10]. As a semireflexive process, the swallowing intensity and muscle movement in the pharyngeal phase are regulated by sensory information from the pharynx. Damage to the primary sensorimotor cortex can lead to decreased cortical activity, and the M1 ischemia model induced by photochemical treatment was used in this study, which explains the decrease in swallowing speed and volume.

Compared with [24, 25] the VFSS, the FEES has greater advantages in assessing vocal fold (VF) symmetry, range of motion, amplitude, speed, and coordination. In our study, a decrease of VF mobility was observed in PSD mice, but the changes in VF structure assessed through the FEES were not significant. It is widely known that VF movement is affected by motor nerve conduction pathways that innervate the internal pharyngeal muscle group [39]. In our study, the ischemic area located in M1 was related to movement, which might affect the weakening of movement of PSD mice (Figure 1k). However, the differences in VF structure and symmetry are not obvious, possibly because the laryngeal motor neuron center is located in the nucleus ambiguus (NA) [40]. The cerebral cortex connects to the laryngeal motor center through bilateral nerve bundles, and only bilateral cortical lesions or bilateral conduction pathway damage can lead to reduced symmetry of motor neurons on the VF [41].

### 4.2 New Vision of the VPM in the Study of Swallowing Function and EA Regulation

Previous studies have reported that any sensory information first forms a synapse in the thalamus before entering the neocortex. Especially VPM plays an important role in different functions, including the processing of sensory information and its transmission to the cortex and sensorimotor control [42, 43]. A recent study has confirmed that the thalamus is a higher‐order brain region that processes sensory information in patients with anorexia nervosa, suggesting that thalamic subregions are a potential target for interventions of swallowing [44]. However, these studies did not provide direct evidence for the mechanism by which the VPM is involved in the swallowing process. In our study, optogenetic and chemogenetic neuromodulation techniques in conjunction with EMG, VFSS, and FEES were used to investigate the role of the VPM in swallowing function and its involvement in the ability of EA‐CV23 to improve swallowing function in PSD. Our results showed that activation and inhibition of the VPM had corresponding improvement and inhibition effects on swallowing function in mice, suggesting that the VPM is an important brain region related to swallowing activity (Figure 4c–e) and is involved in the improvement of swallowing function by EA‐CV23. Interestingly, when chemogenetically inhibiting the VPM in the PSD mice, EA‐CV23 could not reduce the PTT (PSD + VPM^CNO−^ vs. PSD + EA + VPM^CNO−^), which indicated that EA did not work in the case of inhibiting the VPM (Figure e) and the VPM was important for EA‐CV23 treatment for PSD. However, EA‐CV23 still improved ISI after the inhibition of the VPM, suggesting that the VPM is not the only relay station, and that other brain regions, such as the cerebellum and the ventral posterior lateral nucleus (VPL), may be involved in the uploading of sensory information for swallowing [45]. However, this improvement was not statistically different, which to some extent verified the important role of VPM brain regions in swallowing regulation.

Fu et al. [46] have reported that glutamatergic synaptic transmission is an important part of the circuitry between the thalamus and the cortex. Vglut2 is responsible for transporting and regulating glutamate release. It was detected in several nuclei of the thalamus, especially in the VPM, suggesting that the expression of Vgult2 is positively correlated with thalamocortical connections [47]. Our study revealed that the excitatory neurons in the VPM, especially Vglut2, were involved in the regulation of PSD by EA, which was consistent with the above studies and our previous study [10]. We subsequently explored the relationship between the VPM and swallowing function to identify the VPM as a key brain region for PSD treatment with EA‐CV23, which complemented the role of the VPM in swallowing function.

Evidence from animal studies suggests that VPM is involved in the control of swallowing taste transmission and receives an axonal transmission from the NTS [48, 49]. As early as 2006, it was suggested that there is a precise projection from the VPM to the cortex of the posterior central somatosensory area [50]. Recently, Huo et al. established an important role of the cortical‐thalamic‐cortical pathway in brain information processing, suggesting an important role for the thalamus in sensory afferents to S1 [51]. On the basis of the consensus that the NTS and S1 undergo swallowing sensory transmission and processing [52], this study proposes that the NTS‐VPM‐S1 neural circuit is an important sensory afferent pathway for dysphagia. First, we used the HSV‐EGFP virus through paracrine signaling across multiple synapses to track HSV projecting from the NTS. We observed the labeled neurons of the VPM and S1 successively (Figure 5a). However, given the potential transsynaptic properties of the viruses, the possibility of an indirect connection of the VPM‐S1 neural circuit cannot be ruled out. To confirm direct synaptic connections between these brain regions, we combined them with the RV virus system for verification. RV‐transfected neurons were detected in the NTS 21 days after viral expression, indicating the presence of direct synaptic connections in the NTS‐VPM‐S1 neural circuit (Figure 5g). Thus, the present study demonstrated the existence of the NTS‐VPM‐S1 neural circuit.

### 4.3 Role of Sensory Afferent Neural Circuits in Improving the Swallowing Function via EA

Although the mechanism of motor output in the PSD model has been well described in previous studies [10], the peripheral sensory afferent pathways responsible for modulating dysphagia remain unknown. To demonstrate direct synaptic connections, we designed several experiments to verify the function of the NTS‐VPM‐S1 neural circuit in the improvement of swallowing function by EA in PSD mice. The results demonstrated that activation or inhibition of the NTS‐VPM‐S1 neural circuit leads to corresponding enhancement or attenuation of mylohyoid muscle activity in mice (Figure 7c,f). These findings suggested that the NTS‐VPM‐S1 circuit is closely related to swallowing regulation. Notably, the effect of EA on mylohyoid muscle activity was related to the regulation of neural circuits, with activation of neural circuits enhancing the therapeutic effect of EA‐CV23 and inhibition diminishing it. While EMG provides precise neuromuscular recordings, future studies should integrate videofluoroscopic swallow studies for comprehensive functional assessment.

Although the above experiments demonstrated the existence of the NTS‐VPM‐S1 neural circuit and its involvement in the treatment of PSD with EA‐CV23, the existence of other sensory afferent circuits has not been ruled out. Cortical regions of the brain are mainly responsible for the initiation and coordination of swallowing after receiving afferent information, whereas subcortical structures, including the basal ganglia and thalamus, are responsible for the regulation of swallowing through the ganglia‐thalamus‐cortex circuits [53]. Despite chemical inhibition of the VPM brain region and the NTS‐VPM‐S1 neural circuit, the presence of EMG activity in the swallowing muscles of mice suggested that the VPM may not be a unique relay station for sensory stimulation, which in turn indicates that other sensory afferent circuits exist. Therefore, more research is needed to focus on whether sensory signals regulate the swallowing process via other brain regions after EA‐CV23 treatment.

### 4.4 Limitations

First, the PSD model was established by photochemical induction method in this study. We use photosensitive substances to cause endothelial damage and thrombus, leading to the ischemia in M1 of the motor cortex [10, 27]. This process is very similar to the occurrence of human cerebral infarction. Compared with other middle cerebral artery infarction models, the operation is less damaging to the brain tissue, and it has the advantages of simple operation, clear damage area, and can be used for the study of the mechanism of sensori‐motor neural circuits. However, the disadvantages of the model construction are significant; hypoperfusion cannot be equated with infarction, which still differs from those of human stroke, and the depth and precision of research need to be further improved. Additionally, it should be particularly noted that due to interspecies variations and the complexity of human diseases (exemplified by post‐stroke dysphagia in this study), the clinical translation of EA‐CV23 may face significant limitations. Second, in this study, we explored the central mechanism of sensory afferents and verified the regulatory role of the NTS‐VPM‐S1 neural circuit in sensory afferent, which focused on the role of excitatory neurons in the EA‐CV23, but not enough research on inhibitory neurons has been conducted. While EMG provides precise neuromuscular recordings of swallowing, current technical constraints limit our assessment to this modality during neural modulation procedures. Although VFSS and FEES represent the gold standard for clinical swallowing evaluation, their application in animal research remains in its nascent stage. Future studies should focus on (1) developing optimized protocols for implementing VFSS/FEES in basic research settings, and (2) integrating these techniques with wireless optogenetics to enable comprehensive functional assessment while establishing precise correlations between neural circuit mechanisms and swallowing outcomes.

## 5 Conclusion

In conclusion, the NTS‐VPM‐S1 neural circuit was proposed to be an important pathway in dysphagia. We explored how peripheral sensory stimulation is transmitted to the higher sensory cortex through the VPM and promotes the recovery of swallowing function in PSD mice, which provides an experimental basis for the application of EA‐CV23 in the treatment of PSD. These findings not only elucidate the regulatory role of sensory afferents during swallowing but also provide potentially valuable strategies for the treatment of dysphagia.

## Author Contributions

Q.Y., Y.D., Z.D., and H.W. designed all experiments. Y.D., J.H., Q.W., Y.Q.T., and J.C. performed the experiments. J.H., Q.W., F.Z., C.L., X.L., C.L., and Y.D. analyzed the data. Y.D., J.H., Q.W., Y.T., and F.Z., C.L., X.L., H.O., N.X., H.O., R.P., and J.C. contributed to discussion. Q.Y., Z.D., N.X., H.W., and Y.D. wrote the manuscript.

## Ethics Statement

The authors have nothing to report.

## Consent

The authors have nothing to report.

## Conflicts of Interest

The authors declare no conflicts of interest.

## Supporting information


Data S1.

## Data Availability

The data that support the findings of this study are available from the corresponding author upon reasonable request.
