# Supplementary material for: Sensory Afferent Neural Circuits Mediate Electroacupuncture to Improve Swallowing Function in a Post‐Stroke Dysphagia Mouse Model
Source: CNS Neurosci Ther. 2025 Jul 24;31(7):e70514. doi: 10.1111/cns.70514 (PMC12289536; doi:10.1111/cns.70514)
Supplement: Supplementary file 1 — Data S1. [file CNS-31-e70514-s001.docx]

**Supplementary Material**


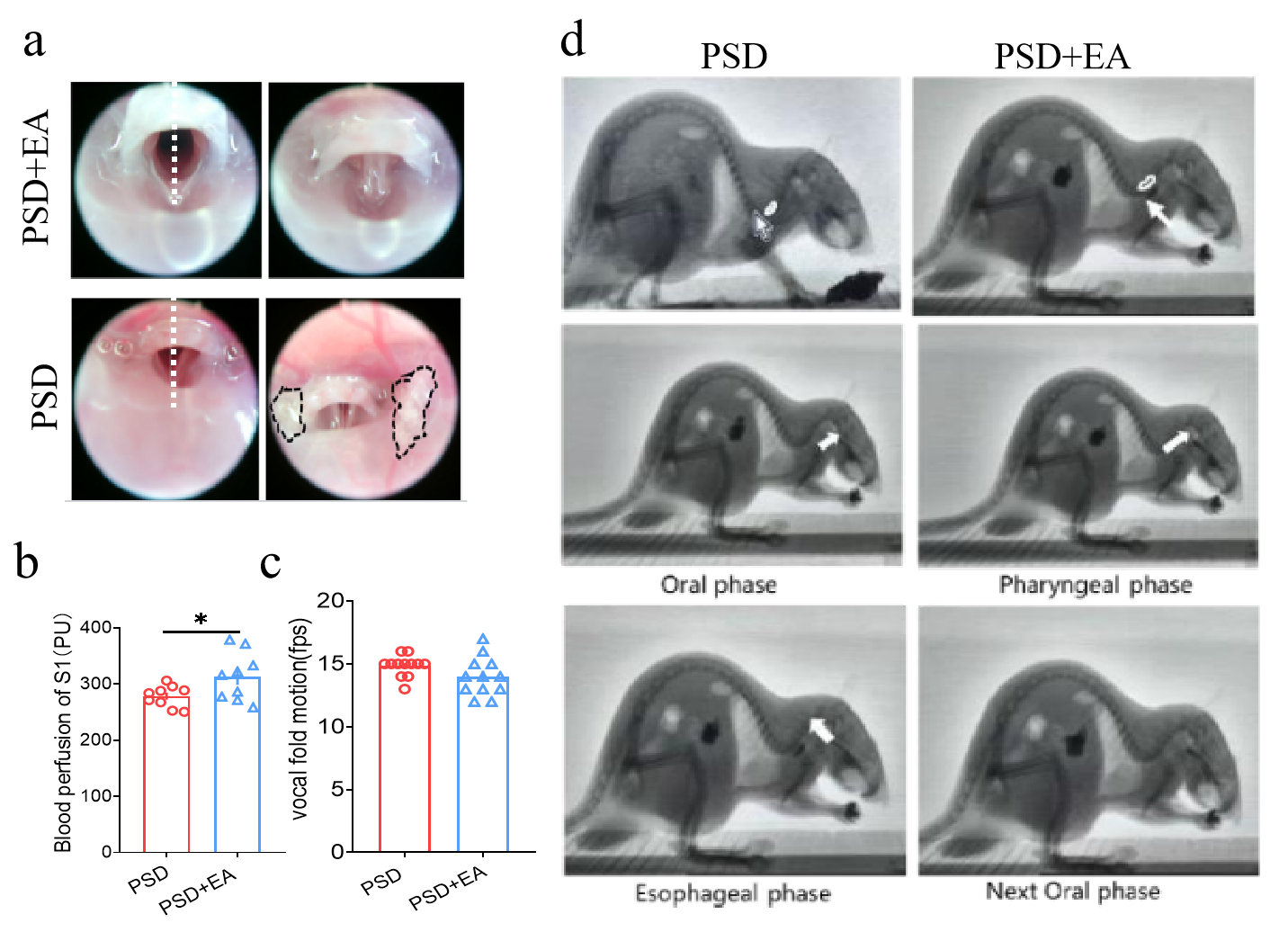


**Sup Figure1. Pharyngeal cavity structure in FEES and swallowing staging in VFSS.**

**a**.Representative diagram of the pharyngeal cavity of mice under laryngoscopy. Upper left: PSD+EA mice with symmetrical abduction of the vocal folds during calm breathing. Upper right: PSD+EA mice breathing calmly with the vocal folds completely closed. Lower left: PSD mice breathing calmly with paralyzed left vocal folds and limited abduction. Lower right: PSD mice with incomplete closure of the vocal folds during calm breathing and a large amount of food remains in the pharyngeal cavity. The vertical white line is the center line and the black box shows the food residues.**b**.The blood perfusion variation of S1 in the PSD group decreased compared with the PSD+EA group (Two-tailed Student's unpaired t-test, t=2.201,n=14, **P*<0.05, PSD vs PSD+EA). **c**.EA-CV23 improves vocal fold cycle movement time (Two-tailed Student's unpaired t-test, t=2.201,n=12, *P＞*0.05, with no significant differences, PSD vs PSD+EA).**d**.Representative diagram of mice swallowing under VFSS.Upper left:PSD mice swallowing one bite size.Upper right:PSD+EA mice swallowing a mouthful.Second row below: Oral phase,pharyngeal phase,esophageal phase and next swallow initiation phase of mice swallowing under VFSS.

**
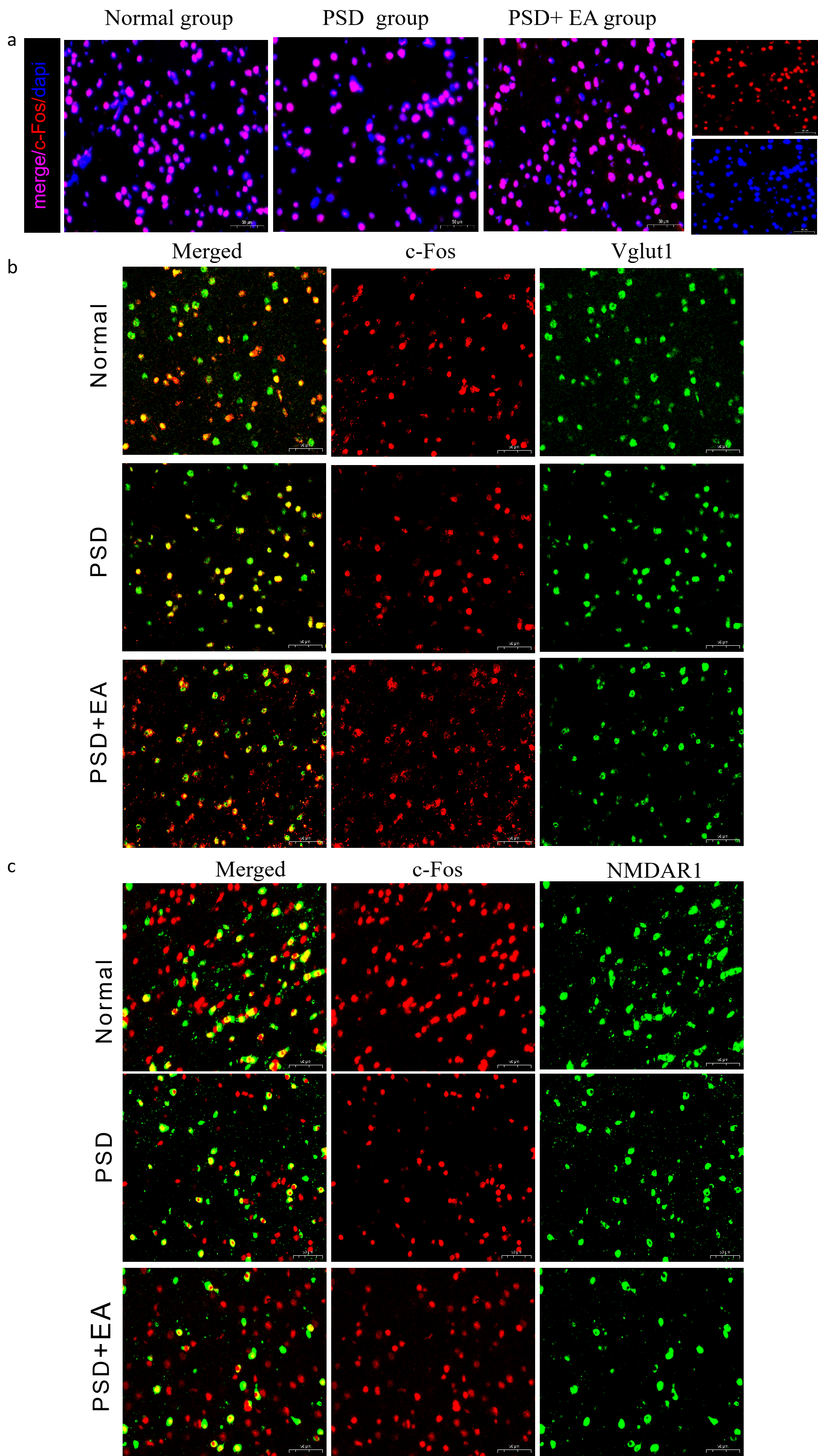
**

**Sup Figure 2. The co-expression of Vglut1, NMDAR1 with c-Fos were detected.**

a. EA-CV23 activates the expression of c-Fos positive neurons in VPM. dapi (blue), c-Fos(red), merger(purple), scale bar: 50um. b. EA-CV23 activates the expression of Vglut1-positive neurons in the VPM. c-Fos (red), Vglut1(green), merger(yellow), scale bar: 50um. c. EA-CV23 activates the expression of NMDAR1-positive neurons in VPM. c-Fos(red), NMDAR1(green), merger(yellow), scale bar: 50um.


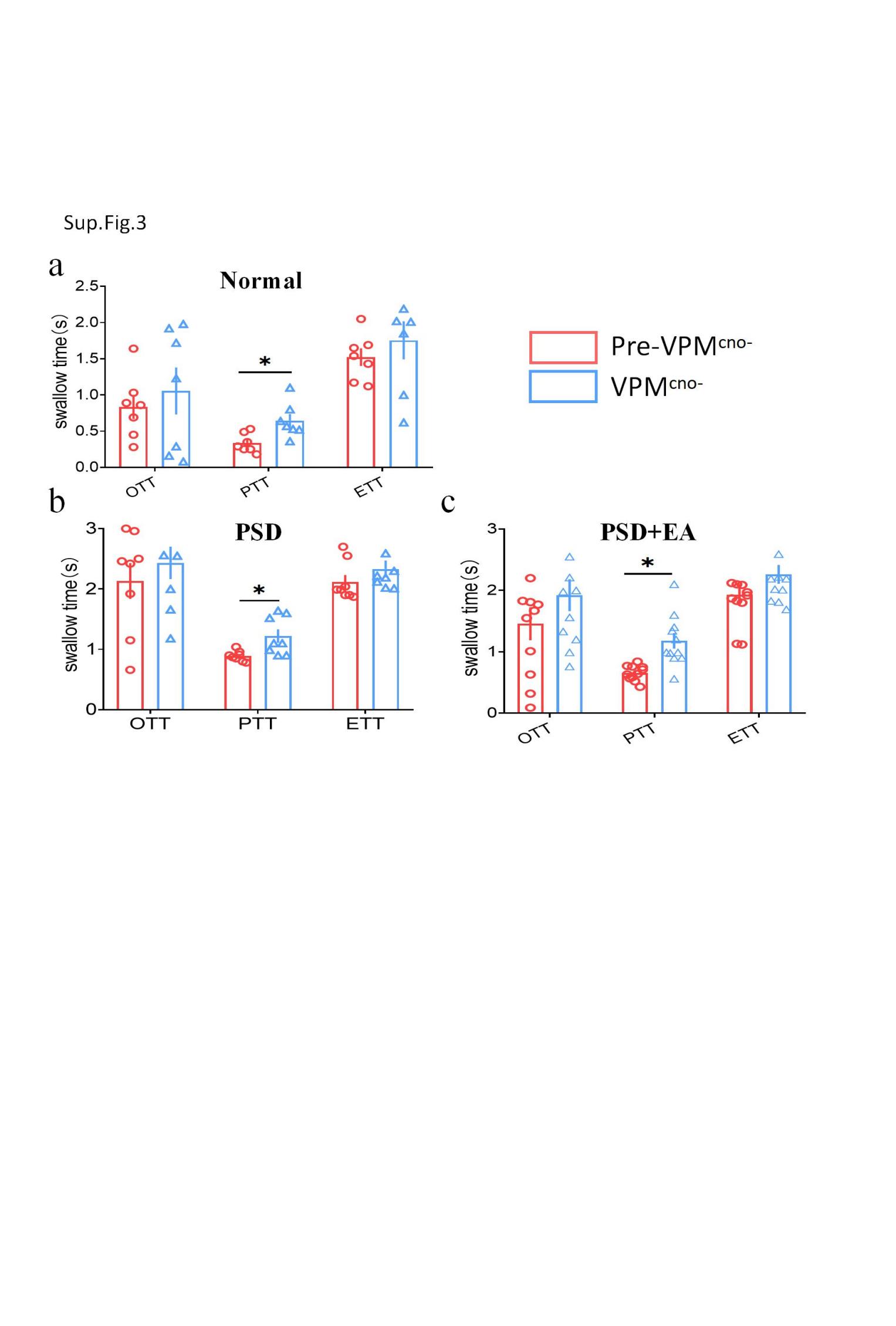


**Sup Figure3. Chemogenetic inhibition of VPM significantly prolongs PTT.**

a. Chemical genetic inhibition of VPM in physiological states significantly prolongs PTT. (Paired t-test,t=-3.067, n=8, *P＜0.05). b. Chemical genetic inhibition of VPM in pathological states significantly prolongs PTT. (Mann Withney Wilcoxon,Z=-2.46, n=8, *P＜0.05). c. Chemical genetic inhibition of VPM significantly prolongs PTT in PSD mice with EA treatment(Mann Withney Wilcoxon,Z=-2.845, n=11, *P＜0.05).


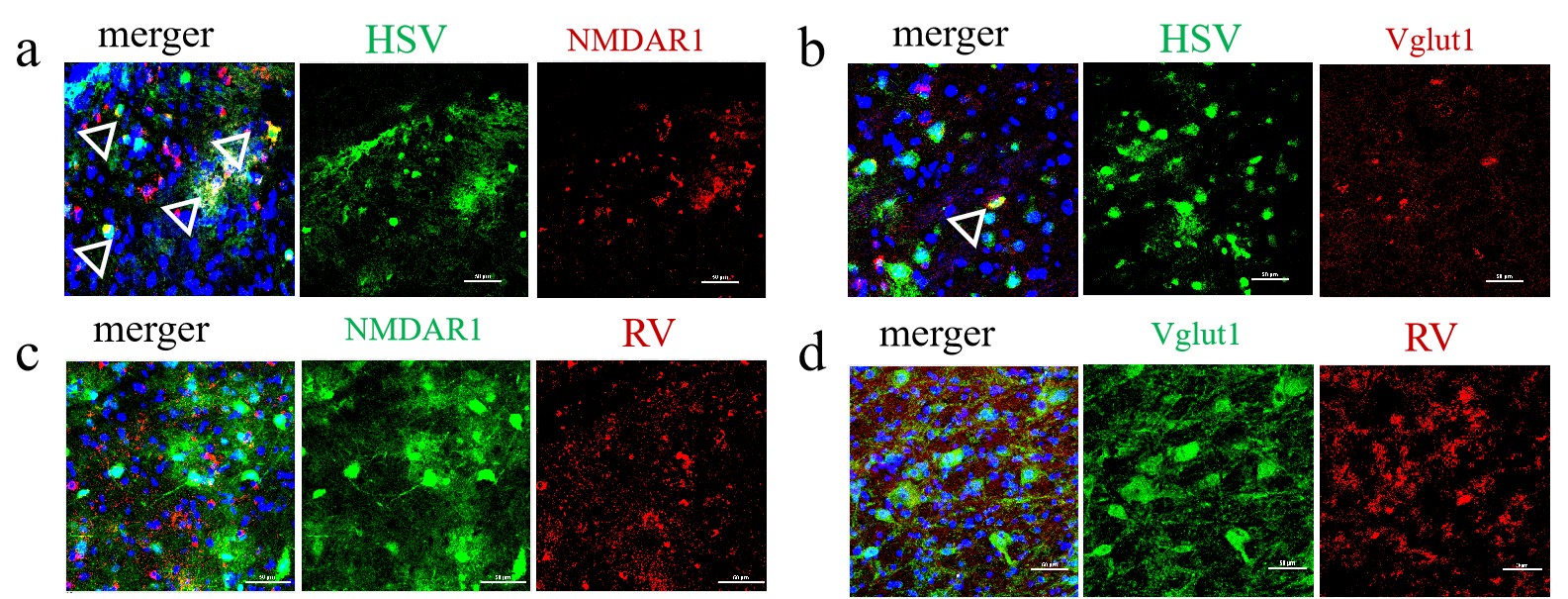


**Sup Figure 4. Direct synaptic connections exist in NTS-VPM-S1.**

**a**. Representative diagram of the co-expression of HSV neurons (green) projected from the NTS to VPM with NMDAR1 (red). merger(yellow), Scale bar, 50 μm. **b**. Representative diagram of the co-expression of HSV neurons (green) projected from the NTS to VPM with Vglut1 (red). merger(yellow), Scale bar, 50 μm. **c**. Representative diagram of the co-expression of RV neurons (red) with NMDAR1 (green) in NTS. Scale bar, 50 μm. **d**. Representative diagram of the co-expression of RV neurons (red) with Vglut1 (green) in NTS.Scale bar, 50 μm.


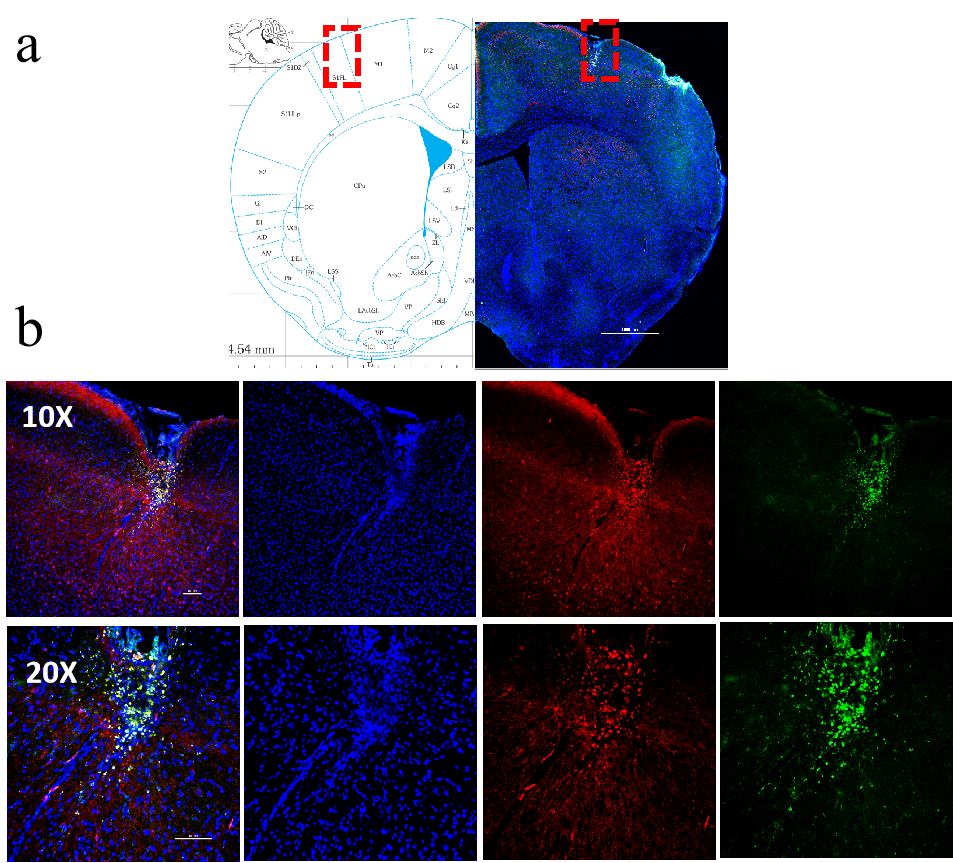


**Sup Figure 5.Representative diagram of electrode buried in S1.**

1. Representative diagram of Multi-Channel Electrodes buried in S1.Electrodes traces are shown in the red line box. **b**. Diagram showing the co-expression of two virus in S1. The up: scale bar : 200 μm. The below : scale bar 100um . AAV2/9-CaMKIIa-ChR2-EGFP (green) and AAV2/9-CaMKIIa-hM4Di-mCherry(red).


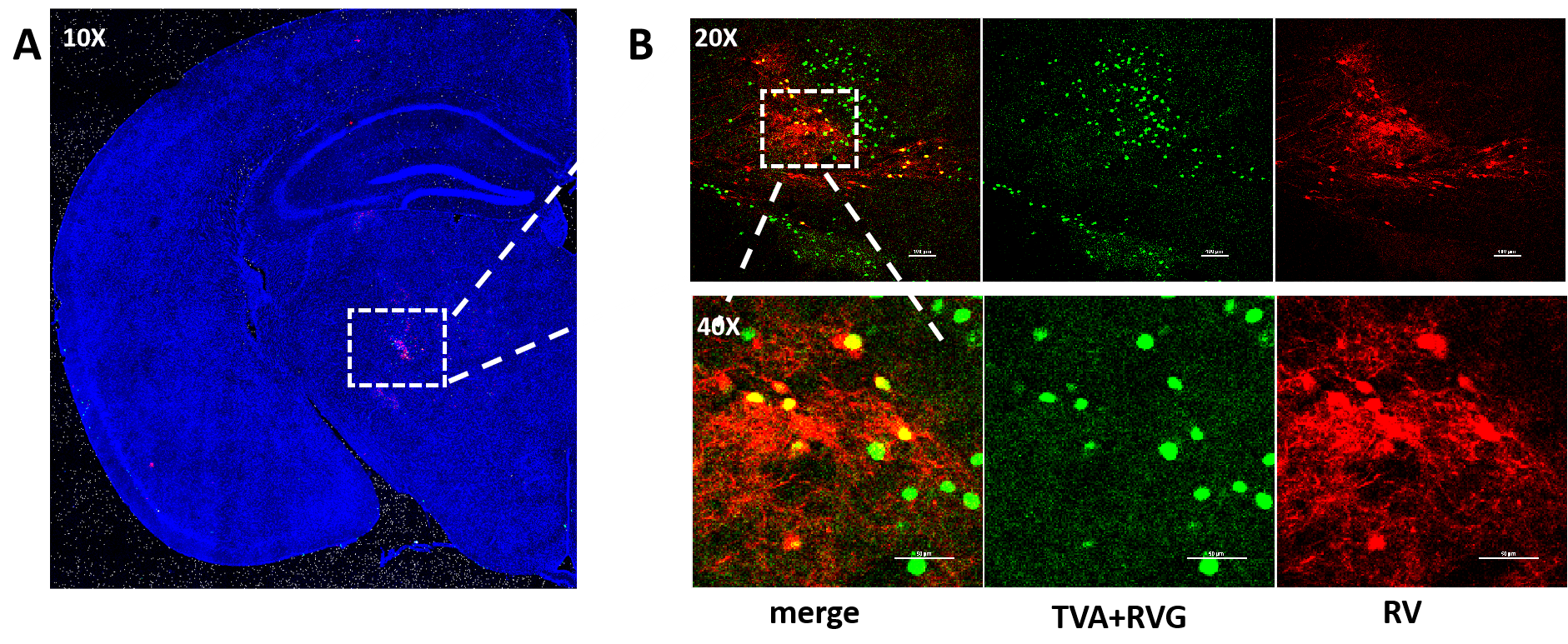

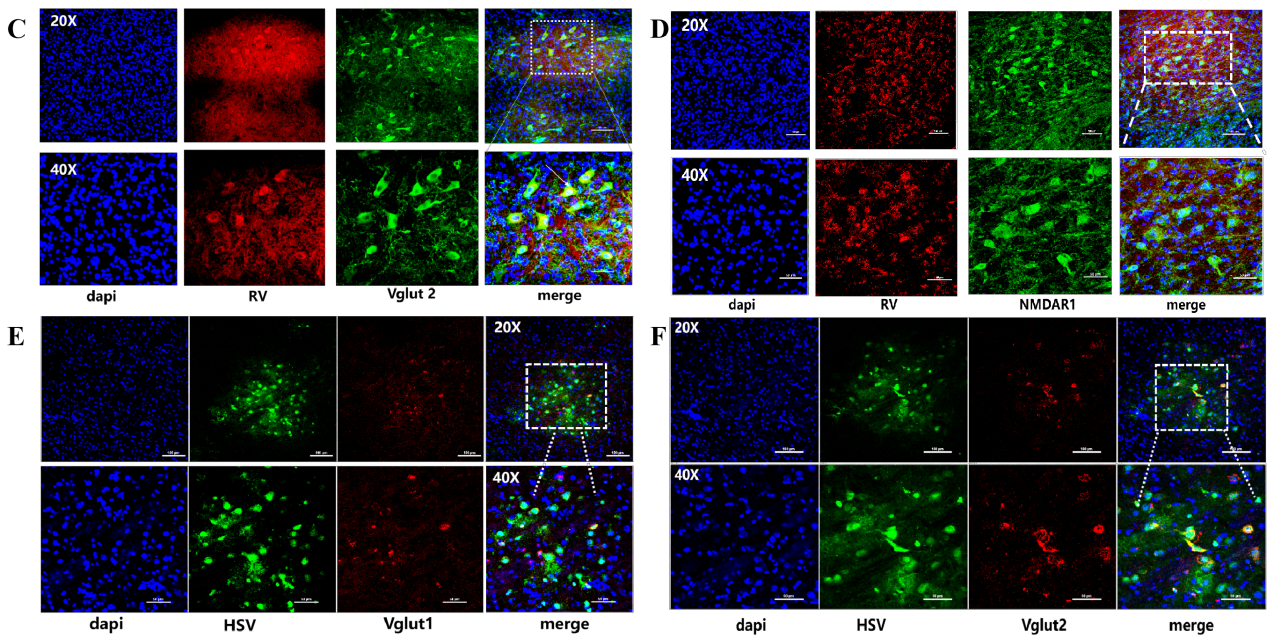


**Sup Figure 6:Representative diagram of Viral tract tracing**

A.Representative diagram of starter neurons expressed in the VPM. red: RV virus, green: helper virus. Scale bar:200 μm (upper panels), 50 μm (lower panels).

B.Representative diagram of starter neurons expressed in the VPM. red: RV virus, green: helper virus. Scale bar:200 μm (upper panels), 50 μm (lower panels).

C.Representative diagram of the co-expression of RV neurons (red) with Vglut2 (green) in NTS. Scale bar:200 μm (upper panels), 50 μm (lower panels).

D.Representative diagram of the co-expression of RV neurons (red) with NMDAR1 (green) in NTS. Scale bar:200 μm (upper panels), 50 μm (lower panels).

E.Representative diagram of the co-expression of HSV neurons (green) projected from the NTS to VPM with Vglut1 (red). Scale bar:200 μm (upper panels), 50 μm (lower panels).

F.Representative diagram of the co-expression of HSV neurons (green) projected from the NTS to VPM with Vglut2 (red). Scale bar:200 μm (upper panels), 50 μm (lower panels).


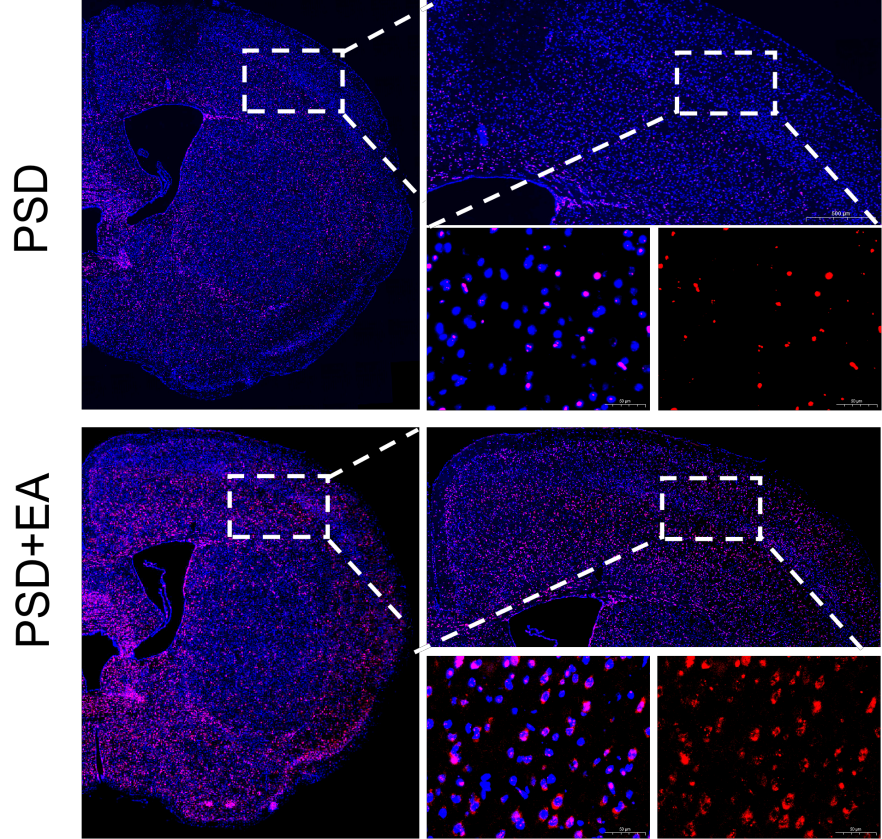


Supplement figure 7: Representative immunofluorescence images showing c-Fos (red) and DAPI (blue) co-expression in S1 cortex of PSD and PSD+EA groups. Scale bars: 1000 μm (overview, left), 200 μm (middle magnification, upper right), and 50 μm (high magnification, lower right).


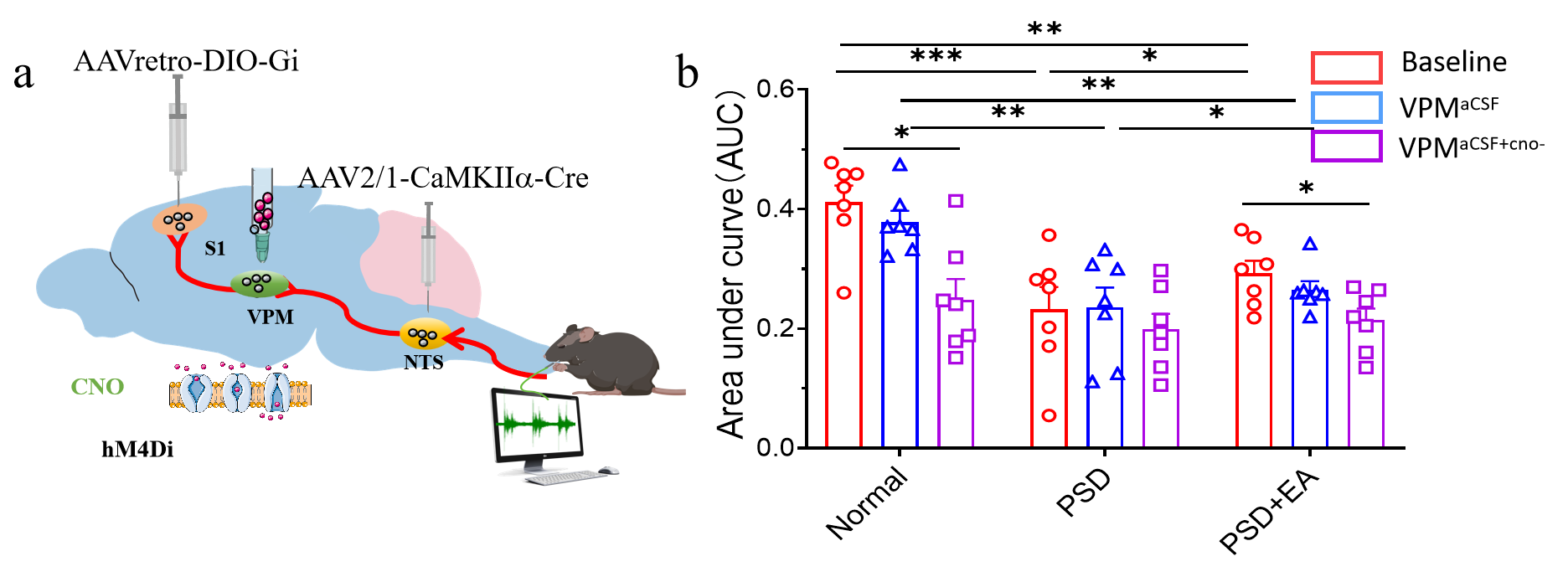


Supplement figure8.Inhibition of the NTS-VPM-S1 neural circuit restricts the therapeutic efficacy of EA-CV23.

1. Schematic diagram showing selective activation of the NTS-VPM-S1 neural circuit. AAV1-CaMKIIα-Cre was injected into NTS and AAV retro DIO-CaMKIIα-Gi was injected into S1 respectively, following with CNO or artificial cerebrospinal fluid (CSF) delivered to the S1 via an implanted tube.
2. The AUC of EMG responses was decreased after inhibitation of the neural circuit among the three groups. (Two-tailed Student's paired t-test, t=3.649, n=7; **P<0.05*, Normal baseline vs. Normal +VPM^aCSF+cno-^. Two-tailed Student's paired t-test, t=3.649, n=7; **P<0.05*, PSD+EA baseline vs. PSD+EA +VPM^aCSF+cno-^. )


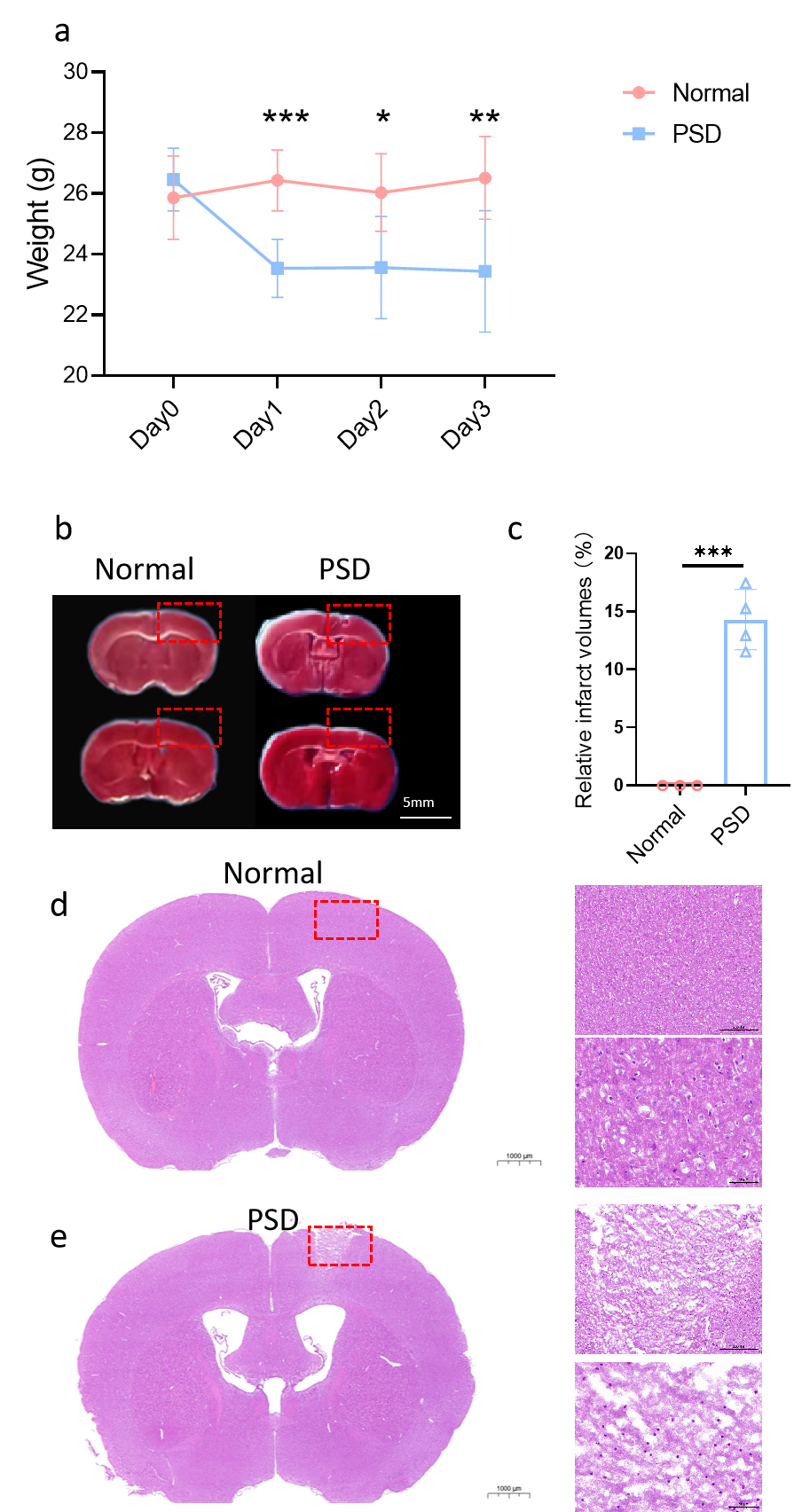


**Sup Figure 9:Representative diagram of TTC and HE**

a. Starting from the first day of modeling, the body weight of PSD model mice significantly decreased compared to that of the normal control group. (Normal: n=6, PSD: n=8). Day 1: ****P* < 0.001, PSD vs. normal group. Day 2: **P* < 0.05, PSD vs. normal group. Day 3: ***P* < 0.005, PSD vs. normal group.)

b. Representative images of TTC staining in the normal and PSD groups. The red box indicates the location of the M1 brain region.

c. After modeling, the infarct area in the M1 region of PSD mice was significantly larger than that in the normal group. (****P* < 0.001, PSD vs. normal group.)

d. Representative images of HE staining in the normal group. Scale bars:1000 μm (overview, left), 200 μm (middle magnification, upper right), and 50 μm (high magnification, lower right).

e. Representative images of HE staining in the PSD group. Scale bars:1000 μm (overview, left), 200 μm (middle magnification, upper right), and 50 μm (high magnification, lower right).
